# Mechanistic Insight into Apoptotic Induction in Human Rhabdomyosarcoma and Breast Adenocarcinoma Cells by *Chnoospora minima*: A Sri Lankan Brown Seaweed

**DOI:** 10.3390/ph14111154

**Published:** 2021-11-12

**Authors:** Thilina Lakmini Gunathilaka, Kulathungage Hiranthi Dilrangi, Pathmasiri Ranasinghe, Kalpa W. Samarakoon, L. Dinithi C. Peiris

**Affiliations:** 1 Department of Zoology, Faculty of Applied Sciences, University of Sri Jayewardenepura, Nugegoda 10250, Sri Lanka; gunathilakathilina2@gmail.com (T.L.G.); hiranthidilrangi001@gmail.com (K.H.D.); 2 Industrial Technology Institute, Halbarawa Gardens, Malabe 10115, Sri Lanka; pathmasiriranasinghe@yahoo.com; 3 Institute for Combinatorial Advanced Research and Education (KDU-CARE), General Sir John Kotelawala Defence University, Ratmalana 10390, Sri Lanka; 4 Department of Zoology/Genetics & Molecular Biology Unit (Center for Biotechnology), Faculty of Applied Sciences, University of Sri Jayewardenepura, Nugegoda 10250, Sri Lanka

**Keywords:** RMS, MCF-7, *Chnoospora minima*, apoptosis, cytotoxicity, caspase pathway, *p53* gene

## Abstract

The current study determined the cytotoxic and apoptotic potential of the polyphenol-rich methanol extract of *Chnoospora minima* (*C. minima*) and its fractions against human breast adenocarcinoma (MCF-7) and rhabdomyosarcoma (RMS) cells. MTT and neutral red assays were used to determine cytotoxicity. The clonogenic assay evaluated the antineoplastic activity, while the apoptotic activity was determined by cellular morphological changes, caspase 3/7 activity, and DNA fragmentation. Morphological alterations in apoptosis were observed by an inverted phase-contrast microscope and Hoechst 33342 staining methods. The total phenolic, flavonoids, alkaloids, and antioxidant activity in the hexane and chloroform fractions were determined, based on their cytotoxic activity. The hexane fraction of *C. minima* effectively reduced the cell growth that is concentration-dependent in human RMS and MCF-7 cell lines. It also exhibited low cytotoxicity on Vero cells. The characteristic cellular and nuclear apoptotic morphological features were observed. A noticeable caspase 3/7 activation and the fragmented DNA were detected only in the hexane fraction treated RMS cells, whereas MCF-7 cells showed low caspase 3/7 activation due to a lack of caspase 3 and no evidence of having a typical ladder pattern of apoptosis. Further analysis revealed that the hexane fraction-treated RMS cells upregulated the *p53* gene twofold (2.72) compared to the *p21* (0.77) gene, whereas in the MCF-7 cells, a 2.21-fold upregulation of *p53* was observed compared to the *p21* (0.64) gene. The hexane fraction exhibited moderate total phenolics, flavonoids, alkaloids content, and antioxidant activity. According to the different antioxidant mechanisms, hexane and chloroform fractions showed the highest antioxidant activities by FRAP and ORAC assays, respectively. GC-MS analysis of hexane fraction revealed the presence of methyl tetradecanoate (38.314%) as the most abundant compound. The study’s findings highlighted that the non-polar compounds present in the hexane fraction of *C. minima* suppressed cell proliferation and induced apoptosis-mediated cell death in RMS and MCF-7 cells, mainly via the activation of the *p53* gene. Hence, the isolation of compounds is warranted. However, more studies are required to understand the mechanistic insights of these observations.

## 1. Introduction

Interestingly, brown algae are one of the fascinating groups of macroalgae that have been a part of the diet of Asian humans for centuries, especially in Japan and Korea [1]. The presence of bioactive compounds contributes to prominent health-promoting effects that can be used in research into anticancer agents [2]. Cancer is the world’s second leading cause of death and is due to uncontrolled cell proliferation [3]. The World Health Organization estimated 24 million cancer diagnoses and 14.5 million cancer-related deaths by 2035 [1]. In Sri Lanka, around 29,604 new cancer patients were identified in 2020, accompanied by 16,691 deaths [2]. Worldwide, breast cancer is the most common cancer in women [4] and the highest prevalence of breast cancer was seen in countries like India, Japan, and other Asian countries in previous years [5]. In Sri Lanka, approximately 3000 new cases are diagnosed each year [6]. Concerning the incidence of cancer in the younger population, rhabdomyosarcoma is one of the most abundant soft tissue sarcomas found in children under ten years old. Every year, 400–500 children in the USA are diagnosed with rhabdomyosarcoma [7]. Due to the high global incidence rate of cancer, it has become increasingly evident that the available therapeutic interventions for different cancers have several side effects [8]. Nausea and vomiting are the most common side effects of synthetic chemical drugs and chemotherapy, other than malabsorption, weight loss, anemia, fatigue, and an increased risk of sepsis due to oral and gastrointestinal mucositis. Similarly, chemotherapy-induced peripheral neuropathy (CIPN) is a common potential toxic effect of cancer treatments [9]. Therefore, lifestyle changes associated with the consumption of healthy foods and nutraceuticals should play a significant role in cancer prevention and management [10]. For this reason, there has been a growing interest in bioactive food components that can be utilized in functional food, nutraceuticals, and pharmaceutical products [11]. For example, carrageenan from *Kappaphycus alvarezii*, *Eucheuma denticulatum*, and *Betaphycusgelatinum* are used in gel formation and coatings in the meat and dairy industry. Species of *Gelidium*, *Gracilaria*, *Hypneas*, and *Gigartina* are used as a source of agar in gel formation and food gums.

Furthermore, the fucans and fucoids in brown algae are used to produce nutraceutical supplements [12]. The polysaccharides extracted from marine algae are used in the bakery industry and various species from *Gracilaria* are utilized to produce food-grade agar due to the presence of 3-linked-β-d-galactopyranose and 4-linked-3,6-anhydro-α-l-galactopyranose [13].

Marine seaweeds are rich in bioactive secondary metabolites, which contribute to their potent antioxidant activity [14]. As oxidative stress plays a vital role in the progression of cancer, natural antioxidants are essential to protect cells’ integrity [15]. Marine brown seaweeds are a potential source of antioxidants and anticancer compounds due to naturally occurring phlorotannins [16,17,18]. Therefore, in the present study, a brown alga, *Chnoospora minima* (Hering 1841), was selected to determine its cytotoxic and apoptotic potential against human rabdomyosarcoma and breast adenocarcinoma cells. As the present study mainly targeted the de-polysaccharide, polyphenolic-rich methanol extract and its fraction of *C. minima*, the factors above may make this work different and significant from other studies.

## 2. Results

### 2.1. Yields of Crude Methanol Extract and Fractions of C. minima

The percentage yield of crude methanol extract from *Chnoospora minima* was based on their dried algae materials. The percentage yield of fractionated extracts (fractions) was calculated using the mass of crude methanol extract, as given in Table 1. The crude methanol extract of *C. minima* presented a maximum yield of 27.2%. The percentage yield of fractionated extract of *C. minima* ranged from 0.1% to 5.3%. The aqueous fraction of *C. minima* had the highest yield (5.3%), while the hexane fraction of *C. minima* had the lowest yield (0.1%).

### 2.2. Cytotoxic Effect of Crude Methanol Extracts and Fractions of C. minima on Human Rhabdomyosarcoma and MCF-7 Cells

The cytotoxic effect of the crude methanol extract of *C. minima* and its fractions was evaluated via human RMS and MCF-7 cells, using the MTT (3-(4, 5-dimethylthiazol-2-cyl)-2, 5-diphenyltetrazolium bromide) and neutral red assays.

#### 2.2.1. MTT Assay

According to the results, hexane (IC_50_RMS: 93.98 ± 1.33; IC_50_MCF-7: 90.58 ± 4.34 μg/mL) and chloroform (IC_50_RMS: 106.94 ± 1.68; IC_50_MCF-7: 97.73 ± 2.92 μg/mL) fractions of *C. minima* exhibited a potent cytotoxic effect on both human rhabdomyosarcoma and MCF-7 cells compared to the standard cycloheximide (IC_50_RMS: 36.17 ± 1.78; IC_50_MCF-7: 28.76 ± 0.55 μg/mL), as given in Table 2. Furthermore, the RMS and MCF-7 cells treated with different extracts and fractions of *C. minima* exhibited a dose-dependent reduction in cell viability (Figure S1). According to the statistical analysis, hexane and chloroform fractions showed significantly high cytotoxic activity (*p* < 0.05) of RMS and MCF-7 cells compared to the other extract/fractions, as shown in Table 2.

Based on these results, the present study selected the hexane and chloroform fractions of *C. minima* for subsequent assays. Further, the cytotoxic activity of hexane and chloroform fractions of *C. minima* on Vero cells was performed to investigate a potential effect of hexane and chloroform fractions of *C. minima* on normal cells’ growth. The hexane fraction exhibited a lower cytotoxic effect on Vero cells (IC_50_: 109.23 μg/mL), while the chloroform fraction (IC_50_: 62.54 μg/mL) exhibited a high cytotoxic effect compared to the human RMS and MCF-7 cells. Similar cytotoxic effects were shown by standard cycloheximide (IC_50_: 29.55 μg/mL) on Vero cells and RMS cells compared to the MCF-7 cells (Table 3). The statistical analysis showed significantly low (*p* < 0.05) cytotoxic activity of Vero cells exerted by hexane and chloroform fractions, compared to the standard that was used (Table 3). Furthermore, hexane and chloroform fraction-treated Vero cells exhibited a dose-dependent cytotoxic effect with standard cycloheximide (Figure S2).

#### 2.2.2. Neutral Red Assay

The neutral red assay was conducted to confirm the cytotoxic activity of active hexane and chloroform fractions of *C. minima* against human RMS and MCF-7 cells. As in the MTT results, the neutral red assay confirmed a decrease in cell viability that was dose-dependent (Figure S3). As presented in Table 4, the hexane fraction-fed MCF-7 cells (IC_50_: 119.46 ± 1.32 μg/mL) exhibited the highest cytotoxic activity when compared with the cycloheximide standard (IC_50_: 27.84 ± 0.33 μg/mL), which were closely similar to the results of the MTT assay. The statistical analysis showed significantly high (*p* < 0.05) cytotoxic activity of RMS and MCF-7 cells exerted by hexane and chloroform fractions, as shown in Table 4.

### 2.3. Hexane and Chloroform Fractions of C. minima-Inhibited Colony Formation

**Figure 1:**
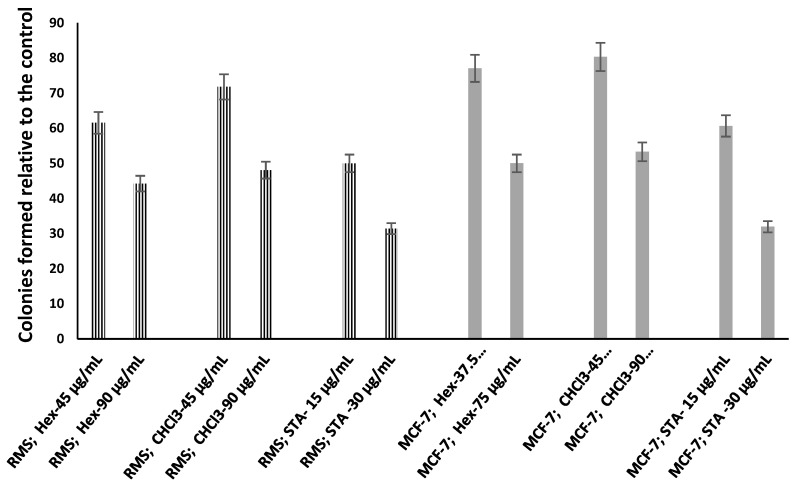
Percentage of colonies formed by RMS and MCF-7 cells after treatment with hexane and chloroform fractions of *C. minima* methanol extract, compared to the standard cycloheximide. Data are expressed as mean ± SD, n = 3.

**Figure 2:**
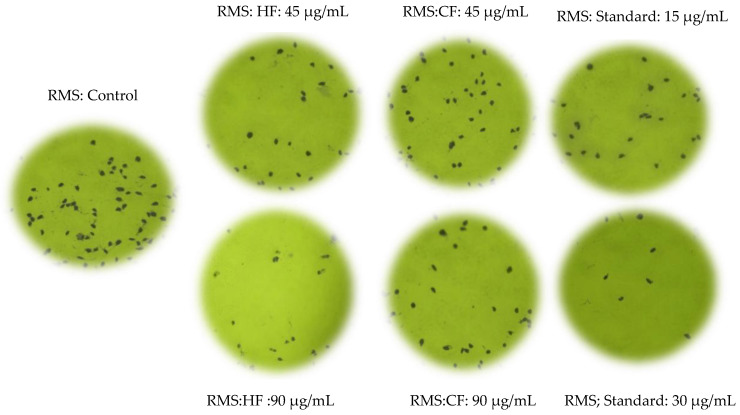
The RMS cells’ colony formation ability, following treatment with the hexane and chloroform fractions at two different concentrations from the standard cycloheximide. HF: hexane fraction; CF: chloroform fraction.

**Figure 3:**
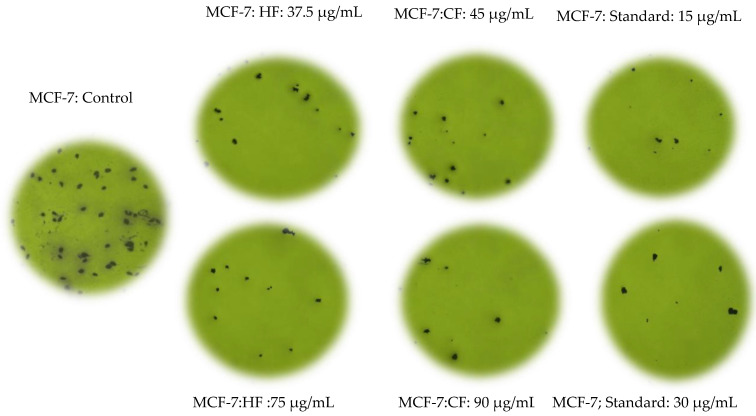
The MCF-7 cells’ colony formation ability following treatment with the hexane and chloroform fractions at two different concentrations from the standard cycloheximide. HF: hexane fraction; CF: chloroform fraction.

The clonogenic cell survival assay determined cells’ ability to proliferate into colonies unrestricted by growth contact inhibition [19]. Two concentrations of hexane and chloroform fractions were selected, based on their cytotoxic potential, to examine the effect on the colony formation capacity of exponentially growing RMS and MCF-7 cells. The percentage of colonies formed by RMS and MCF-7 cells treated with hexane and chloroform fraction is illustrated in Figure 1. Both the RMS (Figure 2) and MCF-7 (Figure 3) cells’ colony formation capacity declined with increasing extract concentrations. The plating efficiency of both RMS and MCF-7 cells decreased after treatments with two concentrations chosen from each fraction. Remarkably, higher plating efficiency (PE) was observed in the hexane fraction-treated RMS (PE: 0.061) and MCF-7 (PE: 0.069) cells at the concentrations of 75 and 90 μg/mL, respectively, as shown in Table 5. The statistical analysis showed a significant difference (*p* < 0.05) between the survival rate of MCF-7 cells exerted by hexane (45 μg/mL) and chloroform (45 μg/mL) fractions, as shown in Table 5.

### 2.4. Hexane and Chloroform Fractions of C. minima Induced Morphological Alterations of Apoptosis in Human Rhabdomyosarcoma and MCF-7 Cells

The morphological characteristics of the apoptosis of human RMS and MCF-7 cells treated with hexane and chloroform fractions of *C. minima* were examined through a phase-contrast inverted microscope and fluorescent (Hoechst stain) staining methods. The morphological alterations in apoptotic cells were observed compared to the untreated cells (control) after incubating the tested hexane and chloroform fractions and standard cycloheximide for 24 h.

#### 2.4.1. Inverted Phase-Contrast Microscope

**Figure 4:**
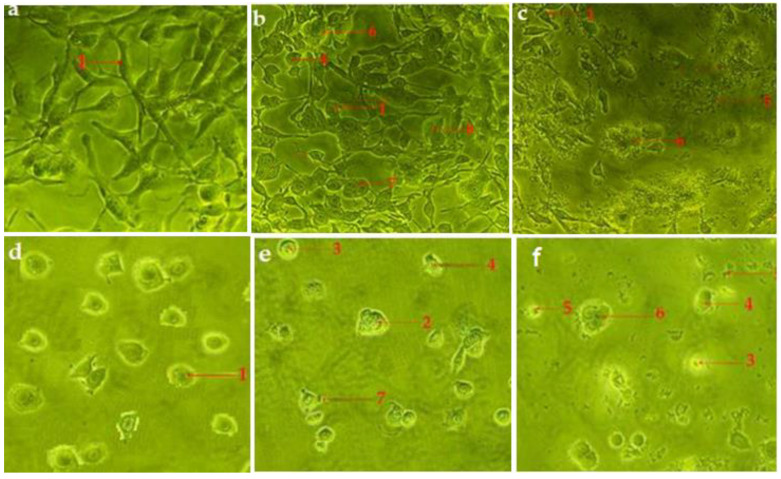
Photomicrographs exhibiting morphological changes of RMS and MCF-7 cancer cells. RMS cells treated with—(**a**): control (untreated cells); (**b**): standard cycloheximide (15 μg/mL); (**c**): standard cycloheximide (30 μg/mL). MCF-7 cells treated with (**d**): control (untreated cells); (**e**): standard cycloheximide (15 μg/mL); (**f**): standard cycloheximide (30 μg/mL) for 24 h and imaged with a phase-contrast microscope (magnification 200×).

According to the cellular observations, the untreated cells (control) of RMS and MCF-7 cells maintained their original morphology, adhesive to the cell culture plate, and they proliferated at a high rate and formed a monolayer growth, with no less than 90% confluence within 28 h (Figure 4a,d).

The RMS (Figure 4b,c) and MCF-7 (Figure 4e,f) cells fed with cycloheximide (15 μg/mL and 30 μg/mL) caused a reduction in cell density and cellular detachment. Furthermore, treated cells showed prominent apoptotic morphological alterations, including cell membrane blebbing, nuclear fragmentation and bulging toward the cell membrane, cellular aggregation to form cell clumps, chromatin condensation, cell shrinkage, or cellular death, and formation of the membrane-bound vesicle at a concentration of 30 μg/mL of standard cycloheximide.

**Figure 5:**
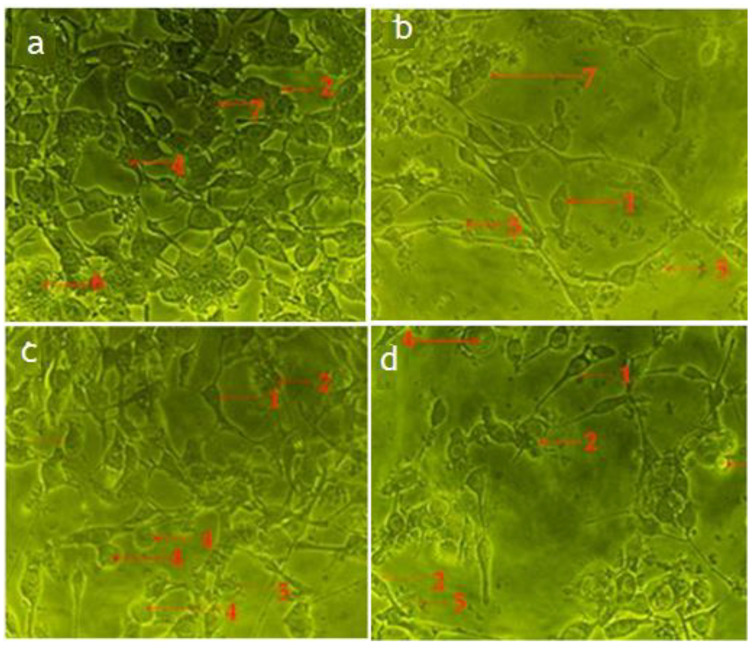
Photomicrograph showing the morphological changes of RMS cells. RMS cells treated with hexane fraction of *C. minima*—(**a**): 37.5μg/mL; (**b**): 75 μg/mL); and chloroform fraction of *C. minima* (**c**): 45 μg/mL; (**d**): 90 μg/mL) for 24 h and imaged with a phase-contrast microscope (magnification 200×).

**Figure 6:**
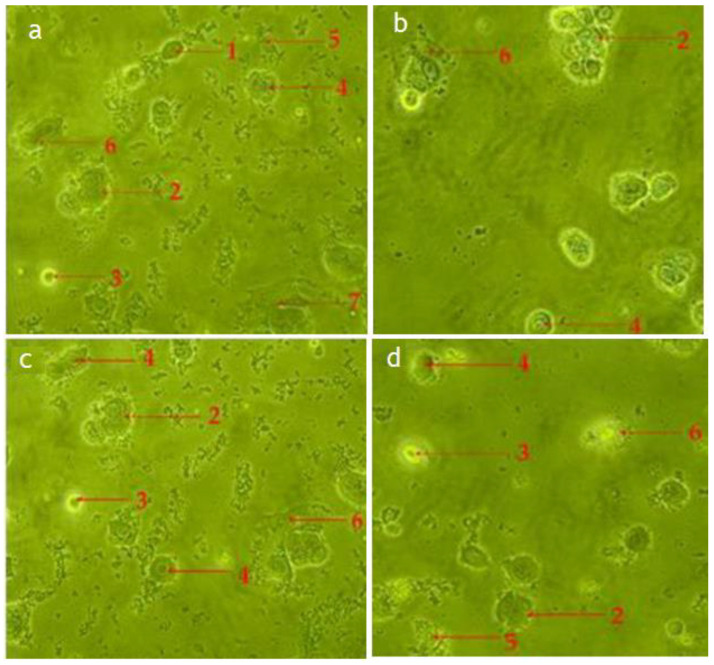
Photomicrograph shows morphological changes of MCF-7 cancer cells. MCF-7 cells treated with hexane fraction of *C. minima*—(**a**): 45 μg/mL; (**b**): 90 μg/mL; and chloroform fraction of *C. minima—*(**c**): 50 μg/mL; (**d**): 100 μg/mL for 24 h and imaged by phase-contrast microscope (magnification 200×).

The apoptotic morphological features observed in Figure 4, Figure 5 and Figure 6 can be explained as follows: (1) cells with normal nuclei; (2) cellular aggregation and formation of cell clumps; (3) chromatin condensation; (4) nuclear fragmentation and bulging toward the cell membrane; (5) cell shrinkage and cellular death; (6) cell membrane blebbing; (7) formation of a membrane-bound vesicle.

Among the tested fractions of *C. minima*, the hexane fraction-treated RMS (Figure 5a,b) and MCF-7 (Figure 6a,b) cells showed noticeable morphological alterations, with weaker cellular adhesion at a concentration of 75 μg/mL and 90 μg/mL, respectively. The cell density of both RMS and MCF-7 cells reduced at 28 h post-treatment, and most of the cells were detached and aggregated in clusters. Similar morphology was observed in the chloroform fraction-treated RMS (Figure 5c,d) and MCF-7 (Figure 6c,d) cells.

#### 2.4.2. Fluorescence Staining (Hochest Staining)

The fluorescence staining method was utilized to observe the morphological changes in cell nuclei in RMS and MCF-7 cells. The blue fluorescent Hoechst 33342 is a cell-permeable nucleic acid dye that is often used to stain the nuclear materials of apoptotic cells to detect chromatin condensation and disintegration.

**Figure 7:**
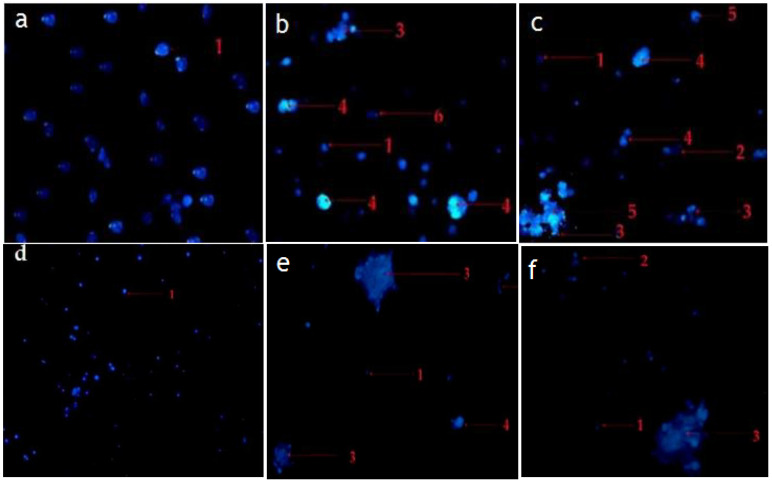
Morphological changes of selected RMS and MCF-7 cancer cells. RMS cells treated with (**a**): control (untreated cells); (**b**): standard cycloheximide (15 μg/mL); (**c**): standard cycloheximide (30 μg/mL). MCF-7 cells treated with (**d**): control (untreated cells); (**e**): standard cycloheximide (15 μg/mL); (**f**): standard cycloheximide (30 μg/mL) for 24 h and imaged via the Hoechst 33342 staining method (magnification 200×).

**Figure 8:**
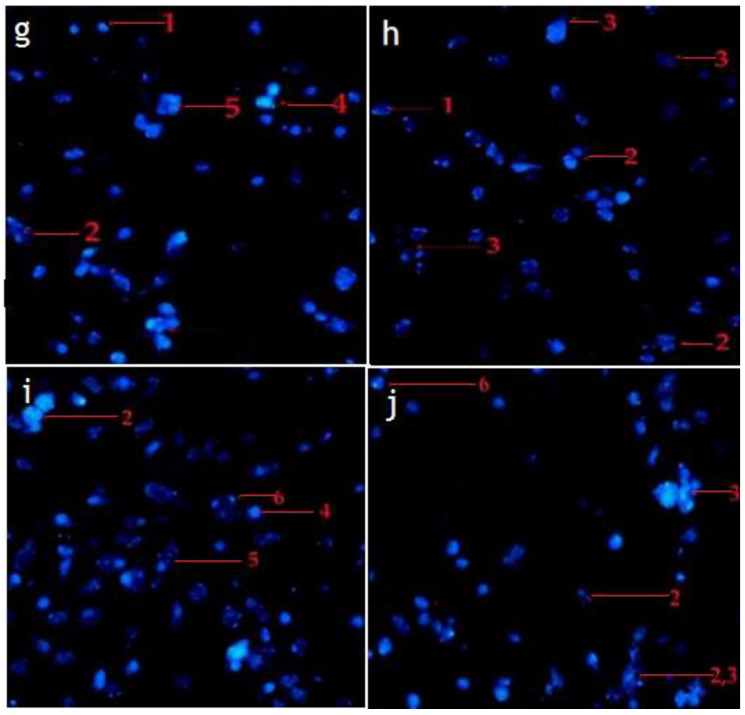
Representative photomicrograph showing the morphological changes of RMS cancer cells. RMS cells treated with hexane fraction of *C. minima* ((**g**): 37.5 μg/mL; (**h**): 75 μg/mL); and chloroform fraction of *C. minima* (**i**): 45 μg/mL; (**j**): 90 μg/mL) for 24 h and imaged by Hoechst 33342 staining method (magnification 200×).

**Figure 9:**
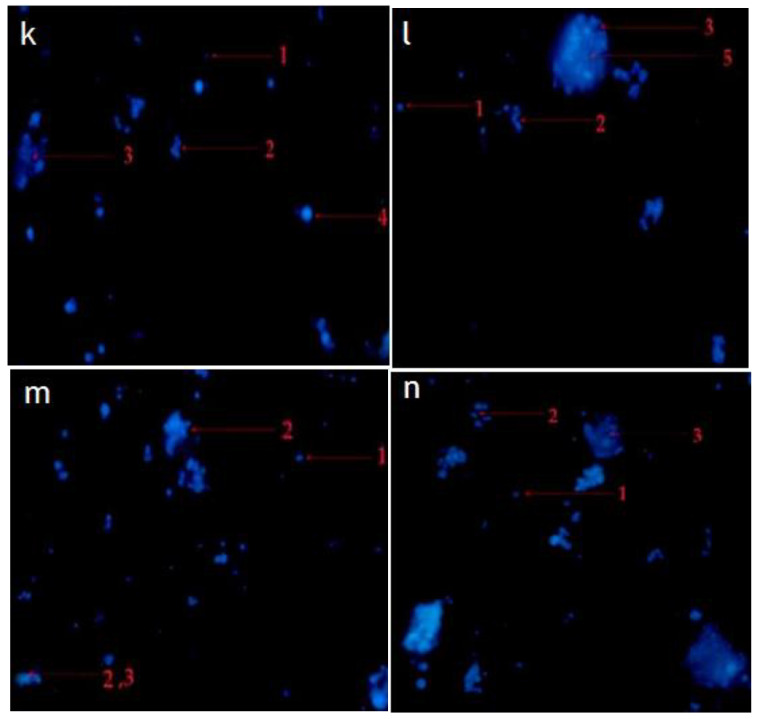
Representative photomicrograph shows morphological changes of MCF-7 cancer cells. MCF-7 cells treated with hexane fraction of *C. minima* ((**k**): 45 μg/mL; (**l**): 90 μg/mL); and chloroform fraction of *C. minima*; (**m**): 50 μg/mL; (**n**): 100 μg/mL for 24 h and imaged by Hoechst 33342 staining method (magnification 200×).

The morphological observation in the untreated cells (control) of RMS and MCF-7 cells showed intact oval-shaped cell nuclei and that they maintained their original nuclear morphology (Figure 7a,d). In contrast, RMS and MCF-7 cells, fed with standard cycloheximide, hexane, and chloroform fraction of *C. minima*, exhibited significant morphological changes compared with untreated (control) cells. Accordingly, cells treated with the standard cycloheximide (Figure 7b,c,e,f), hexane and chloroform fractions of *C. minima* (Figure 8 and Figure 9) showed typical features of apoptosis, including the condensed and fragmented nuclei formation of apoptotic bodies, cell shrinkage, and cell decrement compared to the untreated cells (control). The apoptotic morphological features observed in Figure 7, Figure 8 and Figure 9 can be explained as follows: (1) cells with normal nuclei; (2) formation of an apoptotic cell body; (3) cell membrane blebbing; (4) chromatin condensation; (5) nuclear fragmentation; (6) marginated nucleus.

### 2.5. Hexane and Chloroform Fractions of C. minima Induced Apoptosis and DNA Fragmentation by a Caspase-Dependent Pathway in Human Rhabdomyosarcoma Cells and by a Caspase-Independent Pathway in Human MCF-7 Cells

#### 2.5.1. Caspase 3/7 Activity

The caspase family of proteases is involved in the apoptosis pathway. Therefore, the apoptosis induction of RMS and MCF-7 cells was quantified by measuring caspase 3/7 activity using the *Ac*-*DEVD*-*AFC* substrate, the recognition site of caspases-3 and -7. Staurosporine, a protein kinase inhibitor, was taken as a standard drug or positive control.

**Figure 10:**
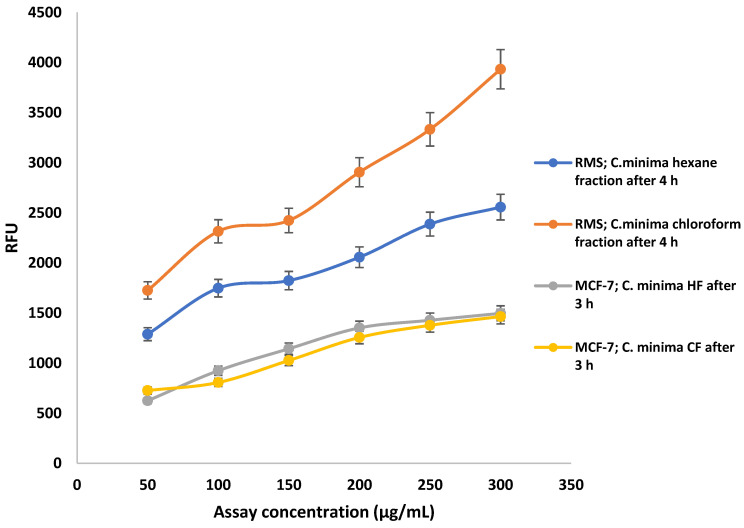
Dose-dependent activation of caspase 3/7 in RMS and MCF-7 cells treated with hexane, chloroform fractions of *C. minima*. Data are expressed as mean ± SD of triplicates.

**Figure 11:**
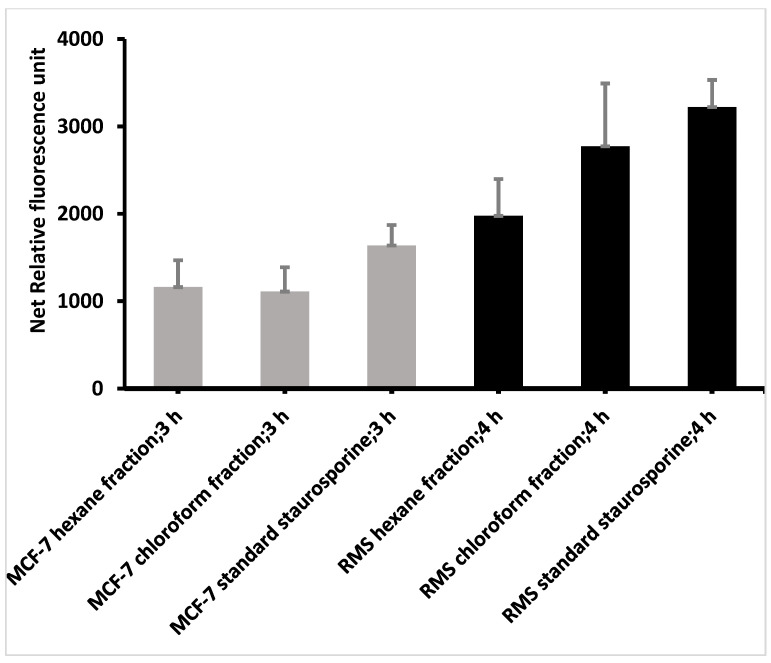
Caspase 3/7 activities of MCF-7 and RMS cells after the treatment with two different concentrations of hexane, chloroform fractions, and the standard staurosporine. In RMS cells, the highest caspase 3/7 activity was observed after 3 h of the caspase treatment, whereas in MCF-7 cells, the highest caspase activity was observed after 3 h of caspase treatment. Data are expressed as mean ± SD, based on triplicates.

After 4 h of caspase treatment, prominent activation of caspase 3/7 was observed in the hexane- and chloroform fraction-treated (24 h) RMS, compared to the standard staurosporine, in a dose-dependent manner (Figure 10). Similarly, higher caspase 3/7 activation was observed in RMS cells treated with standard staurosporine (Figure 11). Besides this, there was no significant increase in caspase 3/7 activity in MCF-7 cells after 24 h treatment with hexane- and chloroform fractions of *C. minima*, which confirm the low activation of caspase 7, as these cells lack caspase 3.

#### 2.5.2. DNA Fragmentation

**Figure 12:**
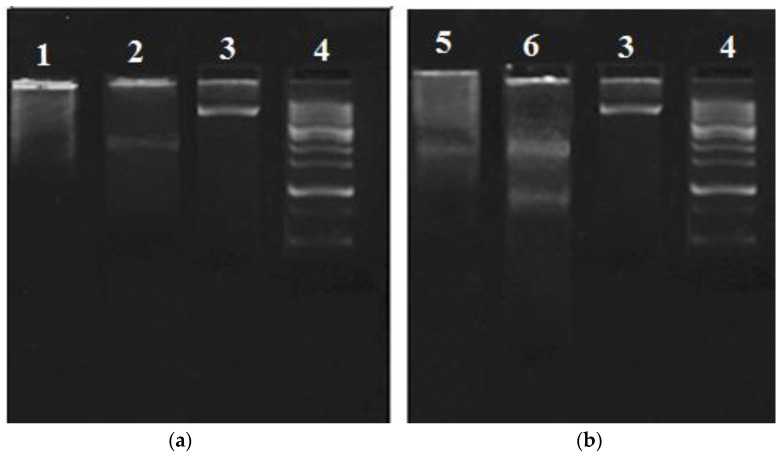
DNA fragmentation shown by agarose gel electrophoresis of MCF-7 (**a**) and RMS cells (**b**) treated with the *C. minima* hexane fraction (RMS-75 μg/mL, MCF-7-90 μg/mL) and standard cycloheximide (RMS-30 μg/mL, MCF-7-30 μg/mL); (1) hexane fraction-treated MCF-7 cells; (2): standard cycloheximide-treated MCF-7 cells; (3) control; (4) DNA ladder; (5) hexane fraction-treated RMS cells; 6. standard cycloheximide-treated RMS cells. Concentrations were selected, based on the IC_50_ values of cytotoxicity.

DNA fragmentation is a hallmark of apoptosis [20]. Therefore, the DNA fragmentation assay was performed to determine the mechanism of cell death, mediated by the highest active *C. minima* hexane fraction and the standard cycloheximide compared to the control, against human RMS and MCF-7 cells. A unique DNA ladder pattern was observed in RMS cells treated with standard cycloheximide (30 μg/mL) and *C. minima* hexane fraction (75 μg/mL) (Figure 12b). In contrast, untreated cells (control) showed no evidence of DNA fragmentation. The MCF-7 cells treated with the hexane fraction did not show any typical ladder pattern (Figure 12a). However, the positive control (cycloheximide-30 μg/mL) showed a band similar to the typical DNA laddering in apoptosis than the untreated cells (Figure 12a).

### 2.6. Hexane Fraction Induces Expression of the p53 Gene More Prominently Than the p21 Gene in RMS and MCF-7 Cells

**Figure 13:**
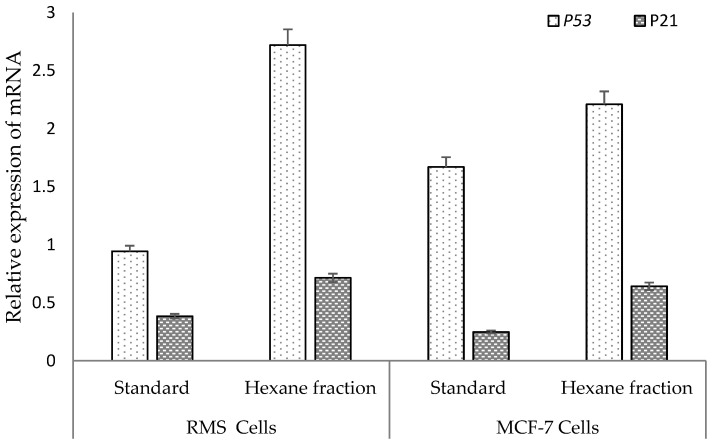
The *p53* and *p21* genes’ relative expression of RMS and MCF-7 treated with hexane fraction (90 μg/mL) and standard cycloheximide (30 μg/mL).

To further explore the effect of the most potent hexane fraction on RMS and MCF-7 cells, *p53* and *p21* mRNA were analyzed via a quantitative polymerase chain reaction (qPCR). The qPCR curves obtained from the Applied Biosystems step one plus real-time PCR systems were Supplementary Material (Figure S4). As shown in Figure 13, hexane fraction (90 μg/mL)-treated RMS cells upregulated the *p53* gene significantly (*p* < 0.05) compared to the standard cycloheximide (30 μg/mL)-treated RMS cells. Similarly, a higher level of *p21* gene expression was detected in hexane fraction-treated RMS cells than the standard cycloheximide-treated cells that were not prominent. The fold change of *p53* and *p21* expression of 30 μg/mL of hexane fraction- and standard cycloheximide (30 μg/mL)-treated RMS cells were 2.72, 0.944 (*p53*) and 0.71, 0.38 (*p21*), respectively.

In MCF-7 cells, a prominent expression of the *p53* gene was detected in hexane fraction- and standard cycloheximide-treated cells. Comparatively, a low induction of *p21* was detected, both in hexane- and standard cycloheximide-treated cells. The fold change of *p53* and *p21* expression of 90 μg/mL of hexane fraction- and standard cycloheximide (30 μg/mL)-treated MCF-7 cells were 2.21, 1.67 (*p53*) and 0.64, 0.25 (*p21*), respectively. Therefore, the results concluded that the hexane fraction-treated RMS cells and MCF-7 cells induce apoptosis via the upregulation of the *p53* gene more prominently, compared to the *p21* gene.

### 2.7. Hexane and Chloroform Fractions Exhibited Moderate Levels of Total Phenolics, Flavonoids, Alkaloids, and Antioxidant Activity

#### 2.7.1. Quantification of Total Phenols, Flavonoids, and Alkaloid Contents

Table 6 depicts the quantified total phenolics, flavonoids, and alkaloids in the hexane and chloroform fractions of *C. minima*. Total phenolic content in both fractions was higher than the flavonoid and alkaloid contents. The chloroform fraction exhibited high phenolic (38.42 ± 3.21 mg GAE/g), flavonoid (3.11 ± 0.51 mg QE/g), and alkaloid (2.79 ± 0.312 mg of PE/g) contents compared to the hexane fraction. According to the statistical analysis, the total phenolic, flavonoid and alkaloid contents of hexane and chloroform fractions exhibited a significant difference (*p* < 0.05), as shown in Table 6.

#### 2.7.2. In Vitro Antioxidant Activity

Antioxidant potential was asserted with 5 different methods, as described in the Section 4. Table 7 shows the antioxidant potential of the hexane and chloroform fractions of *C. minima*.

A dose-dependent radical scavenging ability was observed in both the hexane and chloroform fractions of *C. minima*. The chloroform fraction exhibited the highest antioxidant activity in terms of DPPH radical scavenging activities (IC_50_: 0.75 ± 0.002 mg/mL), ABTS^+^ (IC_50_: 0.12 ± 0.009 mg/mL), chelating activity (IC_50_: 0.93 ± 0.002 mg/mL), ferric reducing antioxidant activity (13.95 ± 1.55 mg TE/g) and oxygen radical absorbance capacity (19.72 ± 2.92 mg TE/g). A significant (*p* < 0.05) positive correlation was observed between iron chelating activity and the total phenolic content of hexane (r = 0.72) and chloroform (r = 0.87) fractions. Similarly, DPPH, ABTS, FICA, FRAP, and ORAC of hexane and chloroform fractions exhibited a significant difference (*p* < 0.05), as shown in Table 7.

### 2.8. Identification of Volatile Components in Potent Anticancer Hexane Fraction by GC-MS

**Figure 14:**
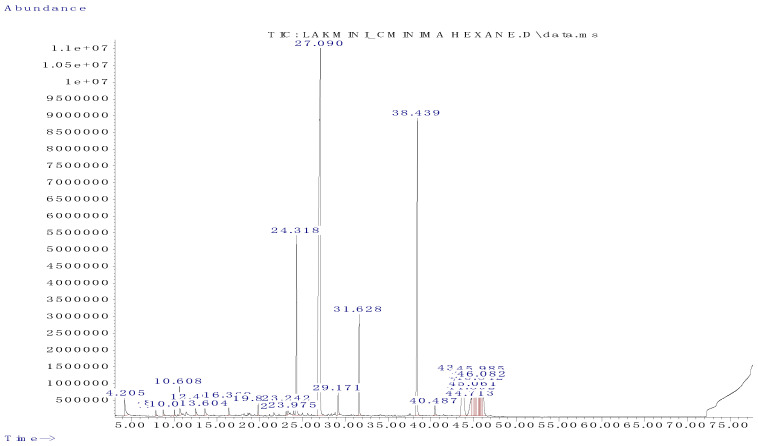
GC-MS chromatogram of hexane fraction of *C. minima*.

Analysis of the total ionic chromatogram (Figure 14) by GC-MS identified four major volatile compounds from the hexane fraction. Relative amounts (%) of the compositions were calculated by peak-area normalization (Table 8). Among the identified compounds, diethyl phthalate and methyl tetradecanoate were identified as compounds with anticancer activity. Dodecanoic acid methyl ester and pentadecanoic acid, 14-methyl-, methyl ester were identified to possess antioxidant activity.

## 3. Discussion

Recently, the utilization of marine seaweeds for different therapeutic purposes has increased significantly due to secondary bioactive metabolites. Among them, brown algae are considered to offer potent anticancer potential. The presence of phlorotannins, sulfated polysaccharides (fucoidan), and fucosterol in brown algae was responsible for its exhibited antioxidant and anticancer activities [25]. Therefore, the present study focused on brown seaweed, *Chnoospora minima*, collected from the Kalpitiya area of Sri Lanka to study its anticancer potential. Human rhabdomyosarcoma (RMS) and human breast adenocarcinoma (MCF-7) cell lines were selected to study anticancer activity.

According to the cytotoxic assays, the hexane fraction of *C. minima* exhibited potent dose-dependent cytotoxic effects toward the RMS and MCF-7 cell lines. Furthermore, the IC_50_ values obtained for MTT and neutral red assays demonstrated that MCF-7 cells are more sensitive to the fractions of *C. minima* compared to the RMS cells. The MTT assay is based on the yellow tetrazolium MTT reagent conversion to purple formazan crystals, using the cellular oxidoreductase enzymes found in mitochondria. In contrast, neutral red dye is a eurhodin stain that accumulates in viable cells’ lysosomes [26]. Parveen and Nadumane (2020) observed that 100 μg/mL *C. minima* exhibited a viability of 63% against MCF-7 cells mL [27]. The current study detected 77.03% cell viability for 100 μg/mL of the methanol extract against MCF-7 cells. The high cytotoxic activity (low cell viability) of the previous study was attributed to the differences in the extraction procedure. The present study used the sonication method for extraction, whereas the previous study used the soxhlet apparatus. Similarly, Parveen and Nadumane (2020) used pure crude methanol extract without including the de-polysaccharide process, whereas the present study only used the polyphenol-rich methanol extract without polysaccharides.

Previously, it was reported that *C. implexa* inhibited the growth of MCF-7 cell lines with an IC_50_ value of 125 μg/mL [28]. Similarly, Khanavi et al. [29] found that crude methanol extract and different fractions of brown algae, including *Sargassum swartzii*, *Colpomenia sinuosa*, and *Cystoseira myrica*, exhibited antiproliferative potential against HT-29, NIH 3T3, T47D, Caco-2, and MDA-MB468 cancer cells. Based on the study, the hexane fraction of *S. swartzii* and *Cystoseira myrica* were most effective against Caco-2 and T47D cells, respectively. Abd Aziz et al. [30] and Pacheco et al. [31] confirmed that fatty acids extracted from macroalgae could suppress the growth of MCF-7 cancer cells. Similar results were demonstrated previously by Hussain et al. [32] with *Phaeodactylum tricornutum*. These results are comparable with the potent cytotoxicity exerted by the hexane fraction of *C. minima* against human RMS and MCF-7 cells in the present study, which may be due to the non-polar structure of cytotoxic compounds. During the de-polysaccharide procedure of the methanol extract, polar compounds can be removed to a certain extent [33]. Most non-polar fatty acid esters were separated into a hexane fraction that significantly impacted the cytotoxic and apoptosis effects on RMS and MCF-7 cells. Furthermore, the GC-MS analysis of the hexane fraction of *C. minima* revealed anticancer compounds, such as diethyl phthalate [22] and methyl tetradecanoate [23], responsible for the observed cytotoxicity and apoptotic activity.

Similarly, the cytotoxic effect of hexane and chloroform fractions of *C. minima* was evaluated using Vero cells as a control to mimic normal mammalian cells. However, hexane fraction-treated Vero cells exhibited a low cytotoxic effect. In contrast, chloroform fraction-treated Vero cells displayed higher cytotoxicity than the human RMS and MCF-7 cells, showing that the hexane fraction exhibited a lower cell killing ability than normal mammalian cells.

Clonogenic cell survival/colony formation assay confirmed the antiproliferative activity of the hexane and chloroform fractions of *C. minima*. The clonogenic assay is a versatile and frequently used tool to quantify reproductive cell survival in vitro. This assay measures the ability of cancer cells to proliferate into colonies without restriction by growth inhibitors [34]. Metastasis is a multi-step process involving the digestion of the extracellular matrix, migration, and colonization of cells to distant sites. During metastasis, malignant tumor cells get detached from the primary site and circulate through the bloodstream to different secondary areas of the body, proliferate into colonies, and eventually form secondary metastatic lesions [35]. Therefore, metastatic colonization is the limiting step in the invasion-metastasis cascade, facilitating the spreading of tumor cells throughout the body. According to the present study, the colony-forming ability of RMS and MCF-7 cells was significantly inhibited by the hexane fractions of *C. minima* (RMS: 75 μg/mL; MCF-7: 90 μg/mL), which confirms the effectiveness of the hexane fraction against metastatic colonization, both in human RMS and MCF-7 cells.

Apoptosis is a form of programmed cell death characterized by different biochemical mechanisms and distinct morphological features. The apoptotic process is an essential homeostatic mechanism for maintaining healthy cells and removing the body’s damaged cells as a defense mechanism. The defects in the apoptotic pathway can lead to the cause of autoimmune diseases and cancer [36]. Therefore, the activation of the apoptotic pathway is one of the main approaches used in cancer therapeutics. In the current study, the apoptotic activity of RMS- and MCF-7-treated methanol extract and fractions of *C. minima* was characterized by cellular morphology, caspase 3/7 activity, and DNA fragmentation.

The induction of apoptosis in RMS and MCF-7 cells by hexane and chloroform fractions of *C. minima* was observed via a phase-contrast inverted microscope. Typical apoptotic morphological alterations, such as chromatin condensation, cell membrane blebbing, DNA fragmentation, the formation of membrane-bound vesicles and cell clumps, and cellular shrinkage, were found in both RMS and MCF-7 cells when compared to the untreated (control) cells. In addition, Hoechst 33342 is used to identify nuclear changes during apoptosis. Hoechst 33342 is a cell-permeable nucleic acid dye used to detect nuclear fragmentation and chromatin condensation during apoptosis [37]. In the Hoechst staining method, live cells were stained uniformly. The apoptotic cells displayed morphological alterations, including cell membrane blebbing, chromatin condensation, nuclear fragmentation, marginated nucleus, cellular shrinkage, and apoptotic body formation in RMS and MCF-7 cells, in contrast to the control cells.

The process of apoptosis is highly complex and occurs in three distinct pathways: the intrinsic (mitochondrial), extrinsic (death receptor), and perforin/granzyme apoptotic pathways, which are ultimately converging on the execution phase of apoptosis [38]. Among the different mechanisms of apoptosis, caspases play a crucial role in apoptosis pathways. Caspases are intracellular cysteine proteases that form a cascade to coordinate the characteristic apoptosis events. All three pathways of apoptosis require specific signals to trigger the activation of caspase 8 (extrinsic path), 9 (intrinsic pathway), and 10 (perforin/granzyme pathway), which eventually activate caspase 3 during the execution phase [39]. There are three executioner caspases, including caspases 3, 6, and 7. Among these, caspase 3 is the most important. Activated caspase 3 cleaves structural proteins, including poly (ADP-ribose) polymerase, endonuclease caspase-activated DNAse with inhibitor (ICAD), and DNA fragmentation factor, resulting in the morphological changes associated with apoptosis. During apoptosis, caspase 3 cleaves the ICAD into CAD (caspase-activated DNAse), which in turn causes chromatin condensation and the cleavage of oligo nucleosomal DNA results in DNA fragments. However, as all three executioner caspases (caspase 3/6/7) possess homologous structures, caspases 6 and 7 can compensate for the function for the loss of caspase 3 during the execution-phase apoptosis [37]. The highest activation of caspase 3/7 was observed in the hexane and chloroform fraction of *C. minima*-treated RMS, which indicates the active apoptotic process in RMS cells. However, a characteristic fragmented DNA ladder pattern was observed in the hexane fraction and standard cycloheximide-treated RMS cells, compared to the untreated (control) cells. These findings agree with those in Parveen and Nadumane’s [27] work. They proved that the methanol extract of *C. minima* induced the activation of caspase 3/7 HeLa and HepG2 cells, which provides evidence for the anticancer potential of the selected brown algae.

In contrast, a low caspase 3/7 in hexane- and chloroform fraction-treated MCF-7 cells was observed. Wang et al. [40] reported that caspase 3 is deficient in MCF-7 cells due to the partial loss of the *CASP-3* gene. Therefore, low caspase 3/7 activity exerted by MCF-7 cells would be due to caspase 7. This point was confirmed by detecting a DNA smear for the hexane fraction-treated MCF-7 cells. However, one light band was detected in cycloheximide-treated MCF-7 cells, which might be due to the activation of executioner caspases 6 and 7 to compensate for the loss of caspase 3 activity during apoptosis. This fact was further supported by the findings by Wolf, who demonstrated that both caspases 3 and 7 induced DNA fragmentation by inactivating ICAD, using an in vitro CAD assay [41]. Therefore, the findings of the present study concluded that the hexane and chloroform fractions of *C. minima* have the potential to induce caspase 7-dependent apoptosis in MCF-7 cells and caspase 3-dependent apoptosis in RMS cells.

Furthermore, the hexane fraction of *C. minima* was selected to evaluate apoptosis-related gene expression (*p53*, *p21*) based on its ability to induce apoptosis in RMS and MCF-7 cells. *p53* is a crucial tumor suppressor that regulates the downstream effector *p21*, a potent inhibitor of cell-cycle kinases. It also functions as a transcription activator of the genes essential for cell cycle arrest, DNA repair, and apoptosis. One of the primary mechanisms underlying the effects of *p53* is how a cell decides to behave, undergoing either cell cycle arrest via *p21* or apoptosis upon *p53* induction. Stresses such as DNA damage, ultraviolet irradiation, hypoxia, oxidative stress, and heat shock induce two sets of *p53*-regulated genes. One set regulates the cell cycle and the other, apoptosis [42]. Our results show that *p53* and *p21* expression in hexane fraction (90 μg/mL)-treated RMS and MCF-7 cells were upregulated, suggesting that *p53* and *p21* mediate the induction of apoptosis in RMS and MCF-7 cells. However, according to the literature, the activated *p53* tumor-suppressor gene stimulates the release of cytochrome C by the mitochondria, which activates the intrinsic mitochondrial apoptosis pathway. Therefore, in the present study, the hexane fraction of *C. minima* induced apoptosis in both RMS and MCF-7 cells via the activation of the intrinsic mitochondrial pathway. This process will be compensated for by other executioner caspases (caspase 6 and 7) in MCF-7 cells, as they lack caspase 3 [43]. The phenolic compounds are secondary metabolites found in seaweeds. The amount of total phenolic content is higher in polar solvents compared to non-polar solvents [44]. In the present study, the chloroform fraction exhibited the highest level of phenols compared to the potent cytotoxic effect exerted by the hexane fraction, which explains the impact of solvent polarity on phenol extraction. As oxidative stress is involved with the pathophysiological conditions of cancer, the combat of oxidative stress through natural antioxidants has increased considerably throughout the world over the last few decades, due to fewer or no side effects [45]. Therefore, in the present study, the antioxidant potential of most potent hexane and chloroform fractions was evaluated in DPPH and ABTS radical scavenging, a ferrous ion chelating assay, ferric reducing antioxidant power, and oxygen radical absorbance capacity. The antiradical power of the natural extracts is widely examined by stable DPPH and ABTS free radicals [46]. Phenols and flavonoids belong to the group of reductones, and they exhibit antioxidant potential by breaking the free radical chain via donating hydrogen atoms. This was evaluated using the ferric reducing antioxidant power [47].

Similarly, the chelating ability of the hexane and chloroform fractions was determined using a ferrozine reagent. The ORAC assay was conducted to determine the peroxyl radical scavenging ability of the natural extract in the presence of fluorescein [48]. Based on the results, the chloroform fraction exhibited somewhat higher antioxidant levels than the hexane fraction, in terms of all antioxidant mechanisms. Therefore, the present study highlighted the low antioxidant potential of non-polar compounds compared to the compounds with intermediate and high polarity. Nawaz et al. (2020) also confirmed the low antioxidant activity of non-polar fractions compared to the intermediate and polar fractions, as most of the antioxidant compounds have a high affinity toward the polar solvents [44]. Therefore, the hexane fraction could only extract lipophilic compounds with a zero-polarity index in the present study. Hence, it showed lower antioxidant potential. Contrastingly, the hexane fraction was identified as the most potent fraction that exhibited anticancer and apoptotic potential regarding human breast cancer and soft tissue cancer cells. Hence, the present study highlights the anticancer potential of hexane fraction, irrespective of its antioxidant activity. This point was further confirmed by a study conducted by Phang et al. (2013). They showed that the hexane fraction of *Alpinia pahangensis* showed lower antioxidant potential, with a potent cytotoxic effect against colon, cervical, breast, and lung cancer cell lines, mainly due to the presence of methyl esters [49]. Therefore, the potent cytotoxic potential of the hexane and chloroform fractions of *C. minima* might be due to the presence of non-polar compounds [50,51], in addition to their antioxidant potential. In the GC-MS analysis, the hexane fraction showed the presence of the significant compounds, methyl tetradecanoate (38.314%) [23], pentadecanoic acid, 14-methyl, and methyl esters (15.999%) [19], dodecanoic acid methyl ester (6.873) [21] and diethyl phthalate (6.309%) [22], which are responsible for its anticancer and antioxidant potential. Therefore, it is highly probable that the toxicity shown by the hexane fraction may be partly due to the presence of non-polar compounds. The cytotoxic effect might be contributed by one or a combination of two or more of these compounds. Their ability to penetrate through the lipid bilayer of the cell membrane causes cells necrosis and loss of cell membrane integrity, leading to cell lysis, or induces apoptotic cell death.

Hence, the present study results signify the cytotoxic and apoptosis effects of RMS and MCF-7 cells exerted by the fatty acid methyl esters that are present in the hexane fraction. This point was further confirmed by Abdelmageed et al. (2017). They found that one of the fatty methyl esters, called oleanolic acid methyl ester, could block cellular proliferation and cell cycle progression, thus inducing apoptosis in prostate cancer cells. They also record that apoptosis is induced via the mitochondrial pathway, where they selectively target cancer cells without affecting the normal cells [52]. Therefore, this mechanism may apply to the present study, as the fatty acid methyl esters present in the hexane fraction exhibited less cytotoxicity on healthy cells than on the RMS and MCF-7 cells. Therefore, it is worth isolating the particular fatty acid methyl esters present in hexane fractions to determine their apoptotic potential and the mechanisms involved, to achieve the maximum utility.

## 4. Materials and Methods

### 4.1. Chemicals and Reagents

High glucose DMEM medium, trypsin-EDTA, penicillin-streptomycin, FBS, 3-(4, 5-dimethylthiazol-2-yl)-2, 5-diphenyl tetrazolium bromide (MTT), crystal violet, agarose, tris-EDTA buffer, gel loading dye, and a DNA ladder were obtained from Sigma-Aldrich (St. Louis, MO, USA). A neutral red cytotoxicity assay kit was purchased from HiMedia Laboratories (Mumbai, India). Caspase Glo 3/7 fluorescence assay kit was obtained from Promega (Madison, WI, USA). All other chemicals were analytical grade. Trolox and EDTA were used as positive controls for antioxidant assays, while cycloheximide and staurosporine were used as positive controls for anticancer assays.

### 4.2. Collection of Algae Sample

*Chnoospora minima* algal samples were collected from the Puttalam district, North Western province (60 40 54.19″ N; 800 80 51.78″ E) during the low tide period (February). The sample was collected after receiving permission from the Department of Wildlife Conservation (permit number WL/3/280/17), and Dr. Kalpa Samarakoon authenticated the *C. minima* sample. The voucher specimen was deposited at the Department of Zoology, Faculty of Applied Sciences, University of Sri Jayewardenepura. The sample was cleaned and crushed into small granules after being dried (Telstar, Frankfurt, Germany) and stored in an airtight container. The voucher specimen of the sample was deposited at the Department of Zoology, Faculty of Applied Sciences, University of Sri Jayewardenepura.

### 4.3. C. minima Extract and Solvent Fractions

The finely powdered sample was extracted into 70% methanol three times using the sonication method, at 25 °C. The polysaccharides present in the sample were precipitated by 70% ethanol (*v*/*w* %—1:25), and the supernatant containing the polyphenols was decanted and kept for further purification [53]. The purified de-polysaccharide methanol extract was partitioned sequentially in an ascending order of polarity using hexane, chloroform, and ethyl acetate. All the experiments were conducted on all four fractions and a part of the dried crude methanol extract [18].

### 4.4. Cell Culture Maintenance

As breast cancer is the most abundant cancer in women worldwide, and soft tissue carcinoma is most prevalent among the child population, the MCF-7 and RMS cell lines were used to determine the efficacy of methanol extract and fractions of *G. edulis* in the present study. Furthermore, Vero cells were used to investigate the potential effect of potent hexane and chloroform fractions on normal cellular growth. The human rhabdomyosarcoma (RMSnd breast adenocarcinoma) (MCF-7) cell lines were obtained from the Department of Biochemistry, University of Colombo, while the Vero cell line was obtained from the Center for Dengue Research, University of Sri Jayewardenepura. The cell lines were grown in DMEM media with 10% FBS and 1% penicillin-streptomycin antibiotic solution. The cells were incubated at 37 °C in a 10% CO_2_ incubator.

### 4.5. Cell Survival Determination

In-vitro cell viability (cytotoxicity) was assessed using neutral red and 3-(4, 5-dimethylthiazol-2-cyl)-2, 5-diphenyltetrazolium bromide) and MTT assays.

#### 4.5.1. 3-(4, 5-Dimethylthiazolyl-2)-2, 5-Diphenyltetrazolium Bromide (MTT Assay)

The cytotoxic potential was evaluated by MTT assay following the method of Mosmann (2008) [54], with some alterations. The viable cell suspensions of 1 × 10^5^ cells/well (RMS, MCF-7, and Vero cells) were seeded in a prepared DMEM medium and incubated in a wet CO_2_ atmosphere (10%) for 24 h at 37 °C. After 24 h, different concentrations of extracts and fractions (10–500 μg/mL) were treated and incubated for another 24 h. After the incubation, well plates were filled with MTT solution (5 mg/mL) dissolved in PBS and incubated for another 4 h at 37 °C. Four hours later, the purplish formazan products were observed, and these were dissolved in acidic isopropanol. The absorbance was recorded at 570 nm using a spectrophotometer (SPECTRA max-Gemini EM, Molecular Devices Inc, (San Francisco, CA, USA). The control (untreated cells) was conducted similarly using the PBS buffer instead of extracts/fractions. Cycloheximide was taken as the standard anticancer drug. The concentration of the sample caused the dose-response curve to quantify 50% of cellular growth inhibition of the cell population (IC_50_).

#### 4.5.2. Neutral Red Assay

The viable cell suspension of 1 × 10^6^ cells/well was seeded in a supplemented DMEM medium and incubated in a CO_2_ incubator at 37 °C. Upon incubation, cells were fed with selected hexane and chloroform fractions (10–500 μg/mL), and further incubation was carried out in a 10% CO_2_ incubator for another 24 h. Cycloheximide was tested as the standard (positive control) anticancer drug, and untreated cells were considered as a negative control. The old medium was aspirated and replaced with 10 μL of the neutral red solution in a fresh medium after incubation. The plates were wrapped with aluminum foil without exposure to the light and were incubated for 2–4 h to allow the viable cells to integrate the neutral red dye into their lysosomes. Cells were observed periodically at intervals, under an inverted microscope, for the presence of stained lysosomes. After incubation, the neutral red fixative solution (100 μL) was used to remove the unbound neutral red dye. Subsequently, it was solubilized with a 1% acetic acid solution in 50% ethanol for around 10 min. The absorbance was reported at a wavelength of 540 nm by spectrophotometer (SPECTRA max-Gemini EM, Molecular Devices Inc., San Francisco, CA, USA). The concentration of the samples inhibited 50% of the cellular growth of the cell population (IC_50_) was detected by linear regression of the dose-response curve [55].

### 4.6. Clonogenic Assay

The ability of cancer cells to proliferate into colonies was measured by a clonogenic cell survival assay. In sterile 6-well plates, the viable cell suspensions were distributed as 1 × 10^3^ cells/well in 6-well plates and incubated for 24 h. The cells were fed with selected doses of hexane and chloroform fractions of *C. minima* and incubated for another 24 h in a CO_2_ incubator. The supernatant was removed, replaced with a fresh medium, and incubated for 5 days in a CO_2_ incubator. The sterile phosphate buffer saline was used to wash the cells and they were then fixed with 75% methanol and 25% acetic acid at room temperature for 5 min. The supernatant was decanted, and the cells were stained with 0.5% crystal violet solution at room temperature and kept for 30 min. The unbound crystal violet stain was further removed using deionized water. The cycloheximide (15 μg/mL and 30 μg/mL) and untreated cells were considered positive control (standard) and negative control. The colonies with more than 50 cells were counted, and plating efficiency (PE) and survival fraction (SF) were calculated [19].

### 4.7. Morphological Alterations in Apoptosis

#### 4.7.1. Inverted Phase-Contrast Microscope

The viable cell suspension of 1 × 10^5^ cells/well was seeded in a supplemented DMEM medium and incubated in a CO_2_ incubator for 24 h. Following incubation, the cells were fed with selected doses of samples/cycloheximide and incubated for another 24 h. The supernatant was drained, and PBS was used to wash the treated cells. The morphological changes of apoptosis were detected by observing an inverted phase-contrast microscope (Nikon Eclipse Ti series, Tokyo, Japan) at 20× magnification. The positive control was cycloheximide, while untreated (control) cells were considered a negative control [55].

#### 4.7.2. Fluorescence Microscope

The viable cell suspension of 1 × 10^5^ cells/well was seeded in a supplemented DMEM medium and incubated in a CO_2_ incubator for 24 h. Following incubation, the old DMEM medium was aspirated. The cells were fed either with two selected concentrations of samples or cycloheximide (positive control) or negative control (untreated cells) with fresh medium and incubated for an additional 24 h. Upon incubation, treated cells were washed with cold phosphate buffer saline (PBS). The Hoechst 33342 (10 μg/mL) dye, prepared in PBS, was used to stain the cells and incubated in a CO_2_ incubator at 37 °C for around 5–10 min. The cells were examined under a fluorescence microscope after removing the excess dye (Nikon Eclipse Ti series, Tokyo, Japan) at 20× magnification [56].

### 4.8. Caspase 3/7 Activity

The Apo-ONE assay for homogenous caspase 3/7 activity of treated cells was evaluated as described by the manufacturer (G7790, Promega, USA). The hexane and chloroform fractions of *C. minima* were dissolved in sterile PBS and examined for 10–500 μg/mL of concentrations. The viable cell suspension of 1 × 10^5^ cells/well was seeded in a supplemented DMEM medium and subjected to overnight incubation in a CO_2_ incubator for 24 h. The cells were fed with various concentrations of extract/fraction and incubated for another 24 h. Before the experiment, the caspase-Glo 3/7 reagent was prepared using substrate and buffer and added to each well at the same volume as the sample, stirred for 30 s. The fluorescence intensity of the reaction mixture was measured at 499 nm of an excitation wavelength and 521 nm of an emission wavelength by spectrophotometer (SPECTRA max-Gemini EM, Molecular Devices Inc., USA) during the incubation period of 30 min–18 h. Staurosporine and cycloheximide are the standards used in the experiment, and untreated cells were considered the negative control [57].

### 4.9. DNA Fragmentation Assay

Viable cells (MCF-7 and RMS) were distributed at a density of 1 × 10^5^ cells/well in 6-well plates and allowed to incubate overnight (24 h). Upon incubation, cells were treated with the required dose of the extracts. As instructed by the manufacturer, cellular DNA was extracted from treated and untreated cells using a TRIzol reagent (T9424, Sigma–Aldrich, St. Louis, MO, USA). The extracted DNA (equal amounts) was separated by electrophoresis in 1.5% agarose gel and examined under UV light, following EB staining [49].

### 4.10. Gene Expression

Viable cells (MCF-7 and RMS) were distributed at a density of 1 × 10^5^ cells/well in 6-well plates and allowed to incubate overnight (24 h). Upon incubation, cells were treated with the required dose of the hexane fraction and standard cycloheximide. As instructed by the manufacturer, cellular RNA was extracted from treated and untreated cells using a TRIzol reagent (T9424, Sigma–Aldrich, St. Louis, MO, USA). The extracted RNA was used to conduct a real-time PCR to evaluate the effect of the most potent hexane fraction on apoptosis-related gene expression in human RMS and MCF-7 cells. *p53* and *p21* were selected to assess the gene expression analysis. The internal reference gene used in the experiment was ß actin. The GoTaq 1-step RT-qPCR system was used to perform real-time PCR as instructed by the manufacturer (A 6020, Promega). The reaction mixture was prepared to the total volume of 10 μL, and the primer concentration was optimized to 200 nM. The primer sequences were tabulated in Table 9.

### 4.11. Quantification of Phenolic, Flavonoid, and Alkaloid Contents

#### 4.11.1. Estimation of Total Polyphenolic Content (TPC)

The Folin–Ciocalteu method was used to determine the TPC of hexane and chloroform fractions. The concentration series of the samples were prepared with deionized water, and 20 µL from the sample was combined with 110 µL of freshly made diluted Folin–Ciocalteu reagent. A ten-percent sodium carbonate solution was prepared and combined with the mixture in each well and kept at room temperature for 30 min. Absorbance was reported at 765 nm. The total polyphenolic content of the extract/fraction was calculated as mg equivalents of gallic acid (standard) per g of dry matter (mg GAE/g) [52].

#### 4.11.2. Estimation of Total Flavonoid Content (TFC)

The aluminum chloride method [58] was used to determine the TFC of the hexane and chloroform fractions. The concentration series of the samples were prepared using methanol. The sample (100 µL) was put into the wells of microplates. A pre-plate reading was recorded to minimize the disturbance due to the color of the sample. The prepared aluminum chloride solution (2%) was added and kept at 37 °C for 10 min of incubation. Absorbance was reported at 415 nm. The TPC content was quantified as mg equivalents of quercetin (standard) per g of dry matter.

#### 4.11.3. Estimation of Total Alkaloid Content (TAC)

The total alkaloid content was quantified using a Dragendorff reagent [59]. Hexane and chloroform fractions (5–10 mg/mL) were prepared with 95% ethanol. The pH of the samples was adjusted to 2–2.55. A hundred microliters of the sample were combined with Dragendorff reagent (200 µL) and centrifuged for 5 min at 5000 rpm to obtain a residue. Following centrifugation, the supernatant was drained, and the residue was purified with 95% ethanol via several washing steps. It was then treated with 1% disodium sulfide solution (200 μL) to obtain a brownish-black precipitate. Subsequently, the supernatant was discarded, and the residue was dissolved entirely in 200 µL of concentrated HNO_3_ and (200 μL) diluted with distilled water. Then, 100 microliters of this solution was pipetted to a new Eppendorf tube and combined with 3% thiourea (500 μL). A wavelength of 460 nm was used to record the absorbance value. The piperine standard curve, Y = 44.861X + 0.032 (R^2^ = 0.992), was used to calculate the total alkaloid content, and it was quantified as mg equivalents of piperine (standard) per g dry matter.

### 4.12. Antioxidant Capacity

#### 4.12.1. DPPH Radical Scavenging Activity

The Blois (1958) method [60] was conducted to determine the DPPH radical scavenging activity. Five different concentrations of selected fractions were made in methanol. The volume of DPPH required for an assay was determined by screening freshly made DPPH solution with methanol. Fifty microliters of each concentration of samples were mixed with methanol (required volume), and the pre-plate reading was recorded at 517 nm. DPPH solution was added to each well and then kept for 15 min at 25 °C in the dark, before measuring the absorbance at 517 nm. The antioxidant activity was quantified as the inhibition percentage, and the dose-response curve was used to calculate the 50% inhibitory concentration (IC_50_) compared to the standard, Trolox.

#### 4.12.2. ABTS^+^ Radical Scavenging Activity

The ABTS^+^ radical scavenging activity assay was conducted by Re et al. (1999) [61]. The ABTS^+^ radical was generated by incubating 10 mg of ABTS tablet in 2.5 mL of 2.5 mM potassium persulfate solution. Five different concentrations of selected fractions were diluted in 50 mM phosphate buffer saline (PBS) (pH 7.4), and 110 µL of PBS was mixed with 50 µL of the sample. The pre-plate reading was reported at 734 nm. Forty microliters (40 µL) of diluted ABTS+ solution was added and kept at room temperature for 10 min for incubation. Absorbance was reported at 734 nm. The scavenging activity was quantified as the inhibition percentage, and the dose-response curve was used to calculate the 50% inhibitory concentration (IC_50_) when compared to the standard, Trolox.

#### 4.12.3. Ferric Reducing Antioxidant Power (FRAP)

The method of Benzie and Szeto (1999) [62] was utilized to evaluate the ferric reducing antioxidant power of the samples. The selected fractions of *C. minima* were prepared in 300 mM acetate butter (pH 3.6) and tested at the range of 1–10 mg/mL. Before use, the working FRAP reagent was prepared using 10 mM 2,4,6-tripyridyl-s-triazine (TPTZ), dissolved in 40 mM HCl, 300 mM acetate buffer (pH-3.6), and 20 mM ferric chloride solution in a 1:10:1 ratio and allowed to incubate at 37 °C for 10 min. The reaction mixture of 20 µL of algae samples, 30 µL of acetate buffer, and 150 µL of FRAP reagent was mixed gently and permitted to incubate at room temperature for 8 min. The absorbance was recorded at 600 nm. The FRAP activity was quantified as mg equivalents of Trolox (standard) per g of dry matter.

#### 4.12.4. Ferrous Iron Chelating Capacity (FICC)

As described by Carter (1971) [63], the chelating capacity of the samples was evaluated by ferrozine reagent in 96-micro well plates (*n* = 4). The selected fractions of *C. minima* were diluted in distilled water and tested at the range of 1–10 mg/mL. Briefly, 100 μL of each extract/fraction, 20 µL of 1 mM ferrous sulfate solution, 40 µL of distilled water were combined, and the absorbance was recorded at 562 nm to obtain the pre-plate reading. Forty microliters (40 µL) of ferrozine reagent (1 mM) were added to initiate the reaction. Upon incubation for 10 min at room temperature, the absorbance was recorded at 562 nm. The chelating capacity of the sample was quantified as the inhibition percentage, and the dose-response curve was used to calculate the 50% inhibitory concentration (IC_50_) compared to the standard EDTA.

#### 4.12.5. Oxygen Radical Absorbance Capacity (ORAC)

The method explained by Ou et al. [64] was used to measure the oxygen radical absorbance capacity (ORAC). The selected fractions of *C. minima* were diluted in 75 mM phosphate buffer solution (PBS) (pH 7.4) and tested at the range of 1–10 mg/mL of concentration. Before the experiment, the fluorescein (4.8 μM) and AAPH (40 mg/mL) solutions were prepared in phosphate buffer (75 mM, pH 7.4). The sample (10 µL) was combined with PBS (40 µL) and fluorescein (100 µL) in a 96-well plate and incubated at 37 °C for 5 min. The reaction was initiated by adding AAPH (50 µL), and fluorescein decay was measured at the excitation wavelength of 494 nm and the emission wavelengths of 535 nm, at intervals of 1 min for a total of 35 min, by a fluorescent microplate reader. Trolox (1.5 and 0.75 μg/mL) was used as the positive control (standard). The control experiment was conducted similarly without the sample. The oxygen radical absorbance capacity was measured as “mg equivalents of Trolox per g of dry matter” using the net area under the curve of fluorescein decay between the control and the extract/fraction.

### 4.13. Gas Chromatography-Mass Spectrometry (GC-MS) Analysis

The GC-MS analysis was performed to identify the potent anticancer-active hexane fraction (Agilent Technologies, Palo Alto, CA, USA). The analysis was conducted using an Agilent GC-MS (7890A GC and 5975C inert MS with Triple-Axis Detector) and HP-5MS capillary column (30 m × 25 μm with a film thickness of 0.25 μm). The carrier gas used for the analysis was helium (flow rate of 1 mL/min), and mass spectra were recorded. Briefly, 1 μL of the prepared sample was introduced into the HP-5MS capillary column. The column was subjected to a range of temperature (70–200 °C) at a 3 °C/min rate and was held at 200 °C for 15 min. The obtained mass spectrum was compared and identified using the NIST 17 library [65].

### 4.14. Statistical Analysis

Each experiment was subjected to statistical analysis using the Microsoft Excel 2016 and Minitab 17 software (Cubic Computing Pvt. Ltd., Bangalore, India). All the experiments were conducted using three replications. The standard deviation (SD) and mean were determined. The significant differences between the samples were determined using a one-way ANOVA. Significant values were defined as those less than 0.05 (*p* < 0.05).

## 5. Conclusions

The current study’s findings provide the cytotoxic and apoptosis effect of hexane and chloroform fractions of *C. minima* on human RMS and MCF cells. The hexane fraction of *C. minima* induced apoptosis via the activation of the *p53* gene and a caspase-dependent pathway in RMS cells, whereas in MCF-7 cells, the activated *p53* gene mainly induced apoptosis. Activated caspase 3/7 induces apoptotic pathways in RMS cells, exhibiting the characteristic morphological alterations of apoptosis and the distinct ladder pattern of DNA. Similarly, the hexane fraction-treated MCF-7 cells showed distinctive apoptotic morphological features of apoptosis even at low caspase 3/7 activation. Therefore, the results highlighted that the non-polar compounds present in the hexane fraction of *C. minima* suppressed cellular proliferation and induced apoptosis-mediated cell death in RMS and MCF-7 cells, mainly via the activation of the *p53* gene, together with its antioxidant activity.

## Figures and Tables

**Table 1:** Yield (%) of crude methanol extracts and fractions of *C. minima*.

Extracts/Fractionated Extracts	% Yield
Crude methanol extract	27.2%
Hexane fraction	0.10%
Chloroform fraction	0.47%
Ethyl acetate fraction	0.19%
Aqueous fraction	5.30%

**Table 2:** Cytotoxic activity (IC_50_) of human RMS and MCF-7 cells treated with the methanol extract of *C. minima* and different fractions compared to the standard, as determined by the MTT assay.

Extract/Fraction	RMS	MCF-7
Crude methanol extract	197.23 ± 5.68	221.15 ± 4.90
Hexane fraction	93.98 ± 1.33 *	90.58 ± 4.34 *
Chloroform fraction	106.94 ± 1.68 *	97.73 ± 2.92 *
Ethyl acetate fraction	235.15 ± 5.45	140.39 ± 1.48
Aqueous fraction	292.65 ± 0.85	162.14 ± 4.44
Standard cycloheximide	36.17 ± 1.78	28.76 ± 0.55

Results are expressed as mean ± SD based on triplicates. *: *p* < 0.05 high cytotoxic activity of RMS and MCF-7 cells exerted by hexane and chloroform fractions compared to other fractions.

**Table 3:** Cytotoxic activity (IC_50_) of Vero cells treated with hexane and chloroform fractions of *C. minima*, compared to the standard cycloheximide.

Extract/Fraction	Cytotoxicity on Vero Cells IC_50_ (μg/mL)
Hexane fraction	109.23 ± 1.05 *
Chloroform fraction	62.54 ± 0.38 *
Standard cycloheximide	29.55 ± 1.56

Results are expressed as mean ± SD n = 3. *: *p* < 0.05; low cytotoxic activity of hexane and chloroform fractions, compared to the standard.

**Table 4:** Cytotoxic activity (IC_50_) of human RMS and MCF-7 cells treated with hexane and chloroform fractions of *C. minima* and the positive control, determined by neutral red assay.

Extract/Fraction	Neutral Red; Cytotoxicity IC_50_ (μg/mL)	
	RMS	MCF-7
Hexane fraction	133.13 ± 4.50 *	119.46 ± 1.32 *
Chloroform fraction	142.09 ± 0.64	135.89 ± 1.16
Standard cycloheximide	32.78 ± 0.91	27.84 ± 0.33

Results are expressed as mean ± SD, n = 3. *: *p* < 0.05; high cytotoxic activity of RMS and MCF-7 cells exerted by the hexane fraction compared to the chloroform fraction.

**Table 5:** Plating efficiency and the surviving fraction of RMS and MCF-7 cells after being treated with hexane and chloroform fractions of *C. minima*, compared to the standard cycloheximide.

	Sample (μg/mL)	Plating Efficiency (PE)	Surviving Fraction (SF)	Survival Rate (%)
**MCF-7 cells**	C.H1: 45	0.096	0.009	61.54 ± 1.23 ^a^
	C.H2: 90	0.069	0.005	44.23 ± 1.08
	C.C1: 45	0.112	0.013	71.79 ± 2.13 ^a^
	C.C2: 90	0.075	0.006	48.08 ± 1.56
	S1: 15	0.078	0.006	50.00 ± 0.98
	S2: 30	0.049	0.002	31.41 ± 1.42
**RMS cells**	C.H1: 37.5	0.094	0.009	77.05 ± 2.54
	C.H2: 75	0.061	0.004	50.00 ± 1.34
	C.C1: 45	0.098	0.010	80.33 ± 3.45
	C.C2: 90	0.065	0.004	53.28 ± 1.45
	S1: 15	0.074	0.005	60.66 ± 1.89
	S2: 30	0.039	0.002	31.97 ± 3.45

Data are expressed as mean ± SD, n = 3. ^a^. refers to the significant difference (*p* < 0.05) of survival rates of MCF-7 cells exerted by hexane and chloroform fractions.

**Table 6:** The quantitative phenolic, flavonoid, and alkaloid contents of hexane and chloroform fractions of *C. minima*.

Extract/Fraction	TPC (mg GAE/g)	TFC (mg QE/g)	Total Alkaloids (mg of PE/g)
Hexane fraction	2.86 ± 0.24	0.23 ± 0.03	1.36 ± 0.69
Chloroform fraction	38.42 ± 3.21 *	3.11 ± 0.51 *	2.79 ± 0.31 *

TPC: total phenolic content; TFC: total flavonoid content; GAE: gallic acid equivalent; QE: quercetin equivalents; PE: piperine equivalent. Data presented as means± standard deviation (*n* = 4). *: *p* < 0.05) TPC, TFC, and total alkaloid contents of the chloroform fraction compared to the hexane fraction.

**Table 7:** IC_50_ values exhibited by hexane and chloroform fractions of *C. minima* against antioxidant activity and activities equivalent to the standards.

Extract/ Fraction	IC_50_ (mg/mL)			Activity Equivalent to Standard (mg TE/g)	
	DPPH	ABTS	FICA	FRAP	ORAC
Hexane fraction	1.88 ± 0.02	4.71 ± 0.31	3.67 ± 0.02	8.57 ± 1.13 **	4.08 ± 1.44 **
Chloroform fraction	0.75 ± 0.002 *	0.12 ± 0.009 *	0.93 ± 0.002 *	13.95 ± 1.55	19.72 ± 2.92
Trolox (standard)	0.011 ± 0.00	0.008 ± 0.00	N/A	N/A	N/A
EDTA (standard)	N/A	N/A	0.019 ± 00	N/A	N/A

Results are expressed as mean ± SD; *n* = 4. *p* < 0.05 compared between two fractions. *: *p* < 0.05) of DPPH, ABTS, and FICA activity of the chloroform fraction compared to the hexane fraction. **: *p* < 0.05—significant high antioxidant activity exhibited by the hexane fraction, compared to the chloroform fraction.

**Table 8:** Active compounds identified in the hexane fraction of *C. minima* by GC-MS analysis.

Retention Time & %	Chemical Name	Nature of the Compound	Molecular Formula	Peak Area (%)	Reported Activity
**24.318**	Dodecanoic acid methyl ester	Fatty acid methyl esters	C_16_H_26_O_2_	6.873%	Antimicrobial Antioxidant [21]
**27.090**	Diethyl phthalate	Phthalate ester	C_12_H_14_O_4_	6.309%	Anticancer Anti-bacterial [22]
**31.623**	Methyl tetradecanoate	Fatty acid methyl esters	C_15_H_30_O_2_	38.314%	Antioxidant Anticancer [23]
**38.439**	Pentadecanoic acid, 14-methyl-, methyl ester	Palmitic acid methyl ester	C_17_H_34_O_2_	15.799%	Antioxidant, antifungal and antimicrobial [24]

**Table 9:** Oligonucleotide primers used for real-time RT-PCR analysis.

Gene	Forward Primer	Reverse Primer
***p21***	5′-CTG-TCA-CAG-GCG-GTT-ATG-AA-3′	3′-TGT-GCT-CA C-TTC-AGG-GTC-AC-5′.
***p53***	5′-GCG-CAC-AGA-GGA-AGA-GA A-TC-3′	5′-CTC-TCG-GAA-CAT-CTC-GAA-GC-3′;
**ß actin**	5′-GTGGGCCGCCCTAGGCACCAG-3′	5′-GGAGGAAGAGGATGCGGCAGT-3′

The fluorescence threshold of the cycle (Ct) represented the crossing point of the amplification curve, and the ΔCt value of the particular gene was calculated as ΔCt = Ct_target gene_ − Ct_ß actin_. The ΔΔCt values were quantified using the formula of ΔΔCt = ΔCt treated − ΔCt untreated. The relative expression level of the target gene in the treated cells was quantified relative to the untreated cells (control) using the formula 2^−ΔΔCT^.

## Data Availability

Data is contained within the article and Supplementary Materials.

## Supplementary Materials

The following are available online at https://www.mdpi.com/article/10.3390/ph14111154/s1, Figure S1: The log-dose cytotoxic effects of RMS treated with (a) standard cycloheximide, (b) hexane fraction, and (c) chloroform fraction of *C. minima* and MCF-7 treated with (d) standard cycloheximide, (e) hexane fraction, and (f) chloroform fraction of *C. minima*. Data are expressed as mean ± SD, n = 3, Figure S2. Cytotoxic assessment of Vero cells following the exposure of (a). hexane fraction; (b) chloroform fraction of *C. minima*; (c). standard cycloheximide., Figure S3. Cytotoxic assessment of human RMS cells by neural red assay following the exposure of (a) cycloheximide (standard), (b) hexane fraction (c) chloroform fraction of *C. minima* methanol extract, and MCF-7 cells following the exposure of (d) cycloheximide (standard), (e) hexane fraction (f) chloroform fraction of *C. minima* methanol extract. Data are expressed as mean ± SD; n = 3., Figure S4. qPCR curves. (a) qPCR curves obtained for ß-actin gene in treated RMS cells, (b) qPCR curves obtained for *p53* gene in treated RMS cells, (c) qPCR curves obtained for *p21* gene in treated RMS cells, (d) qPCR curves obtained for ß-actin gene in treated MCF-7 cells, (e) qPCR curves obtained for p53 gene in treated MCF-7 cells, (f) qPCR curves obtained for *p21* gene in treated MCF-7 cells.

## Abbreviations

RMS	Human rhabdomyosarcoma
MCF-7	Human breast adenocarcinoma
MTT	3-(4, 5-dimethyl thiazolyl-2)-2, 5-diphenyltetrazolium bromide
DNA	Deoxyribonucleic acid
FBS	Fetal bovine serum
DMEM	Dulbecco’s Modified Eagle’s medium
PBS	Phosphate buffered saline
GC-MS	Gas chromatography-mass spectrometry
TPC	Total phenolic content
TFC	Total flavonoid content
TAC	Total alkaloid content
DPPH	2,2-diphenyl-1-picrylhydrazyl
ABTS	2,2′-azino-bis(3-ethylbenzothiazoline-6-sulfonic acid
ORAC	Oxygen radical absorbance capacity
FRAP	ferric reducing antioxidant power
FICC	ferrous iron chelating capacity
