# Supplementary material for: Mechanistic Insight into Apoptotic Induction in Human Rhabdomyosarcoma and Breast Adenocarcinoma Cells by Chnoospora minima: A Sri Lankan Brown Seaweed
Source: Pharmaceuticals (Basel). 2021 Nov 12;14(11):1154. doi: 10.3390/ph14111154 (PMC8622854; doi:10.3390/ph14111154)
Supplement: Supplementary file 1 [file pharmaceuticals-14-01154-s001.zip › pharmaceuticals-1395953-supplementary.pdf]

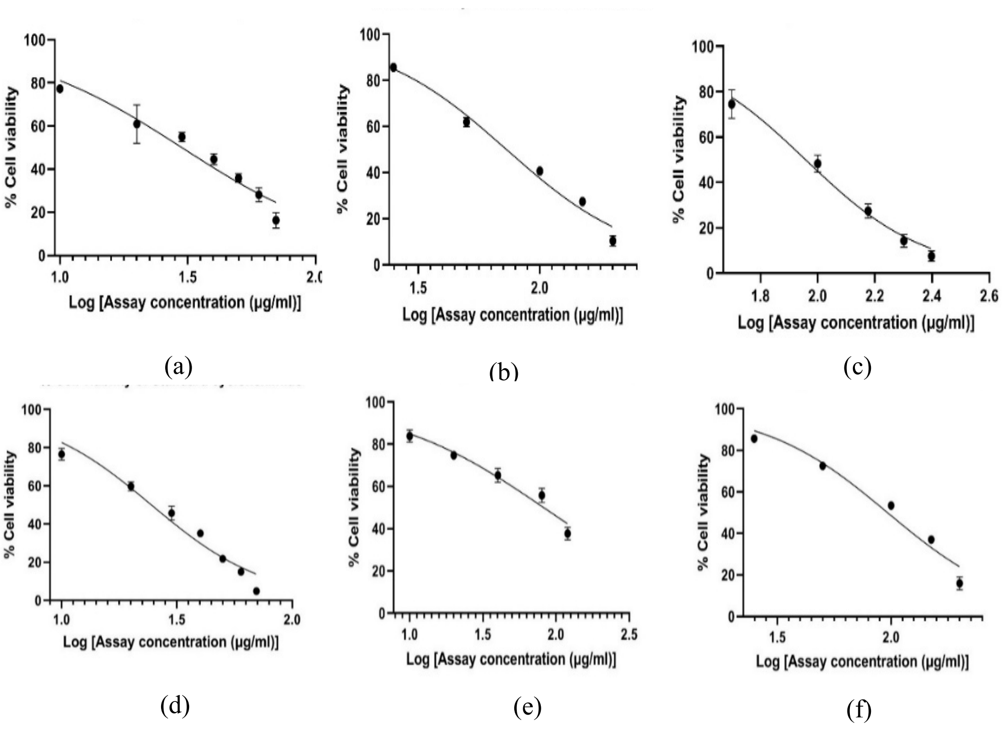

Figure S1. The log-dose cytotoxic effects of RMS treated with (a) standard cycloheximide, (b) hexane fraction, and (c) chloroform fraction of *C. minima* and MCF-7 treated with (d) standard cycloheximide, (e) hexane fraction, and (f) chloroform fraction of *C. minima*. Data are expressed as mean ± SD of triplicate

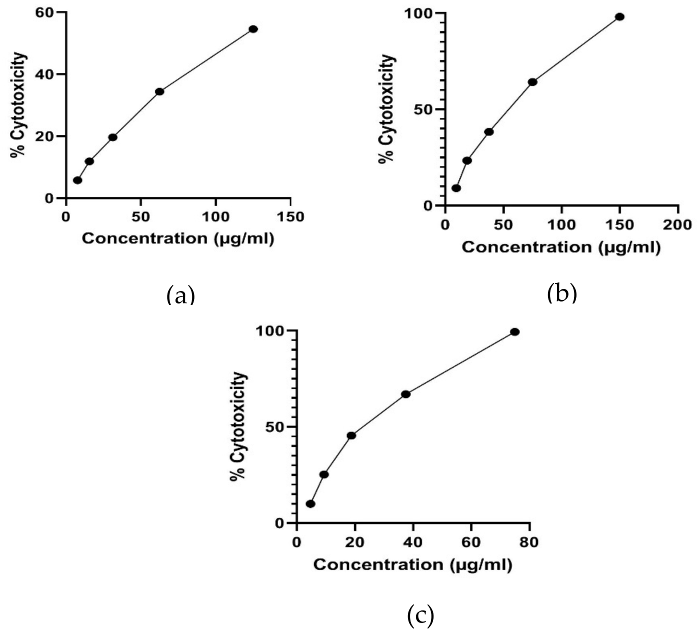

Figure S2.Cytotoxic assessment of Vero cells following the exposure of (a). hexane fraction; (b) chloroform fraction of C.minima; (c). standard cycloheximide.

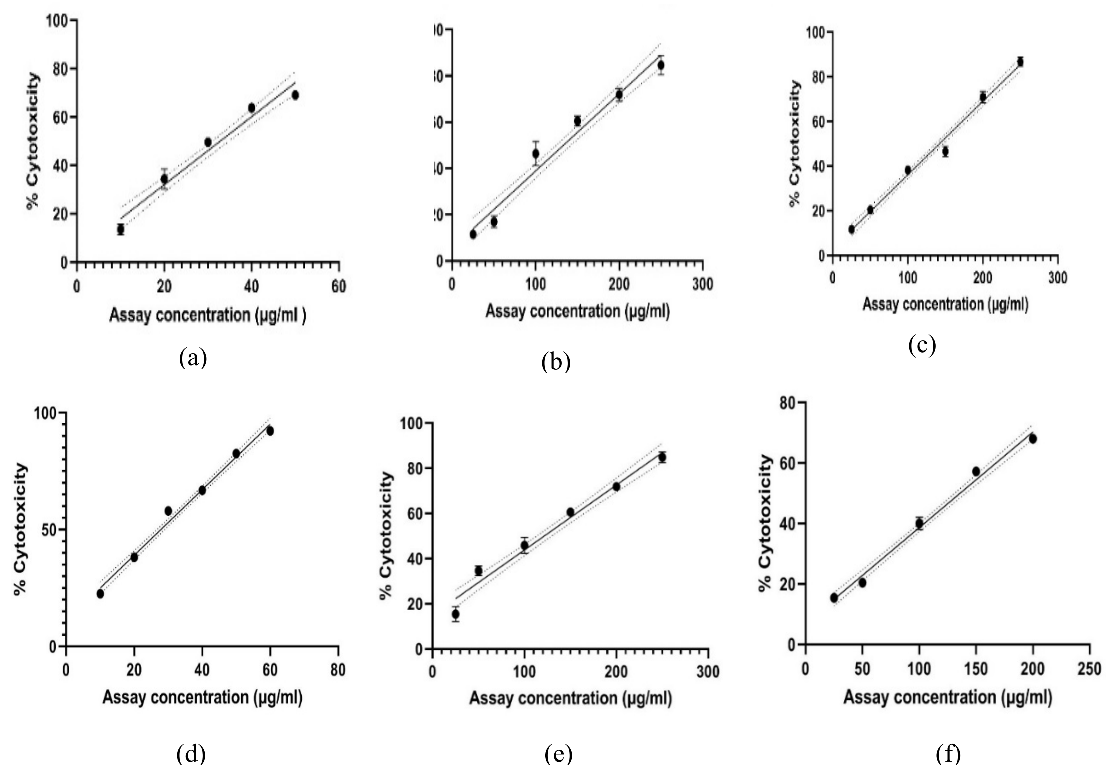

Figure S3.Cytotoxic assessment of human RMS cells by neural red assay following the exposure of (a) cycloheximide (standard), (b) hexane fraction (c) chloroform fraction of *C .minima* methanol extract, and MCF-7 cells following the exposure of (d) cycloheximide (standard), (e) hexane fraction (f) chloroform fraction of *C .minima* methanol extract. Data are expressed as mean ± SD; n=3.

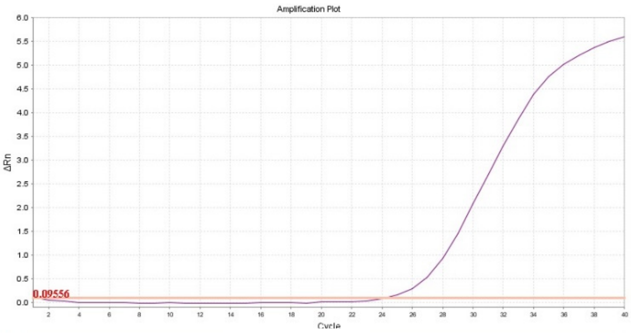

Control

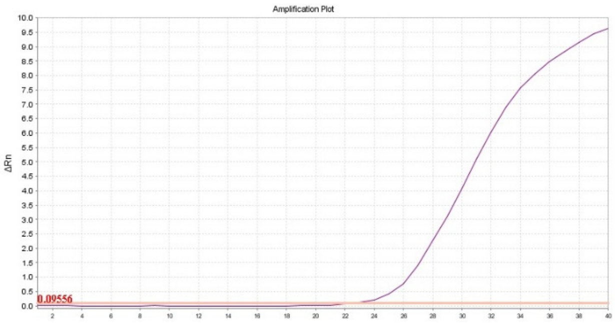

Standard

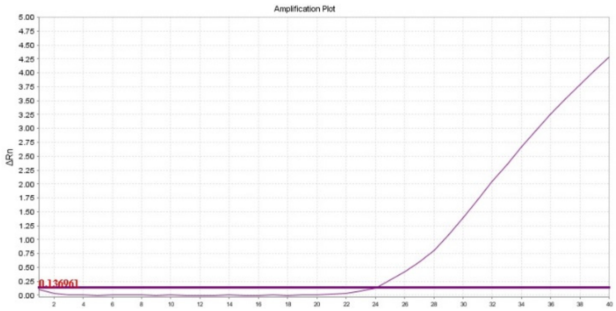

Hexane fraction

(a) qPCR curves obtained for ß-actin gene in treated RMS cells

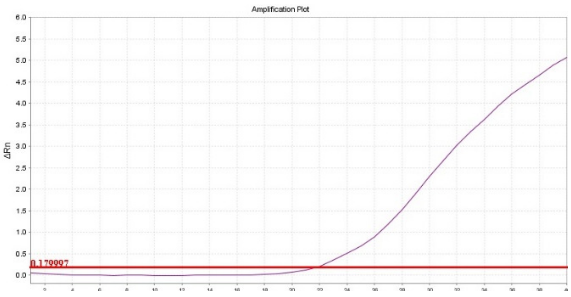

Control

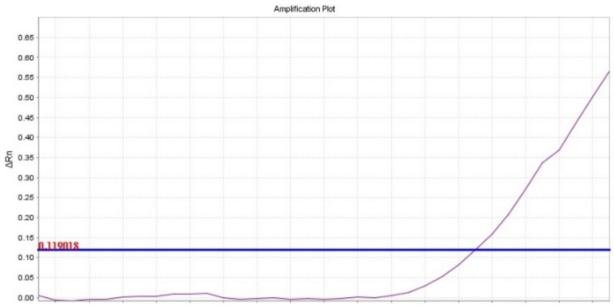

Standard

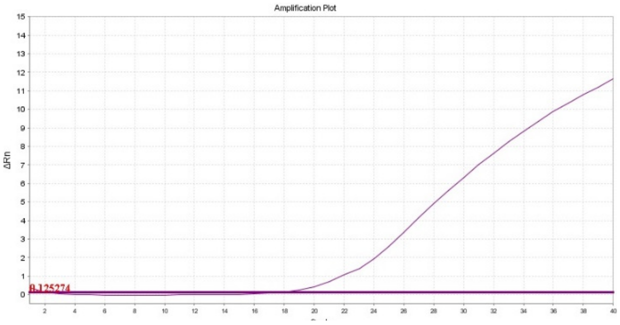

Hexane fraction

(b) qPCR curves obtained for p53 gene in treated RMS cells

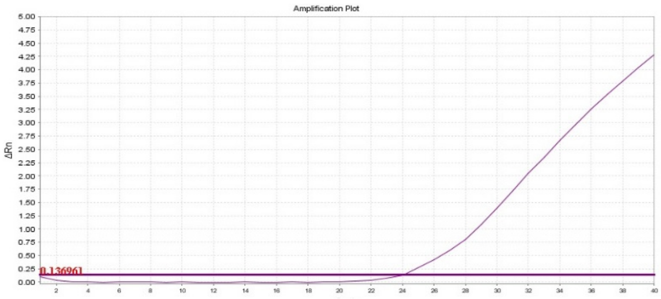

Control

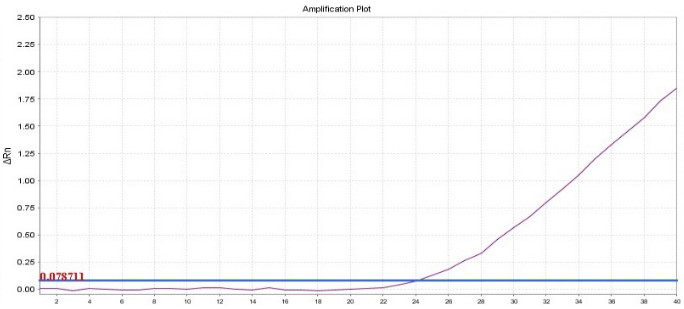

Standard

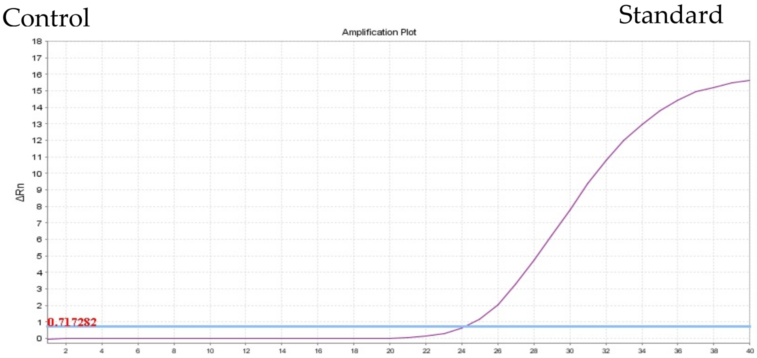

Hexane fraction

(c) qPCR curves obtained for p21 gene in treated RMS cells

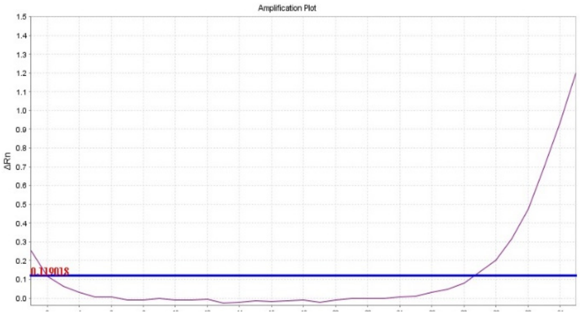

Control

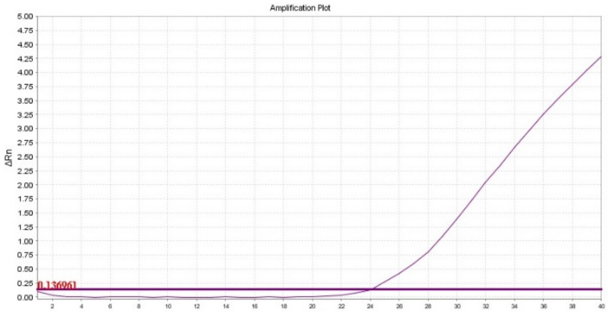

Standard

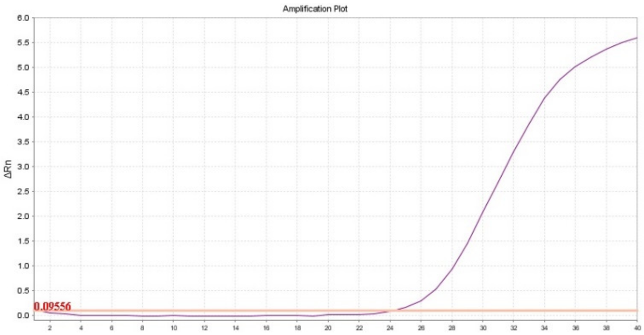

Hexane fraction

(d) qPCR curves obtained for ß-actin gene in treated MCF-7 cells

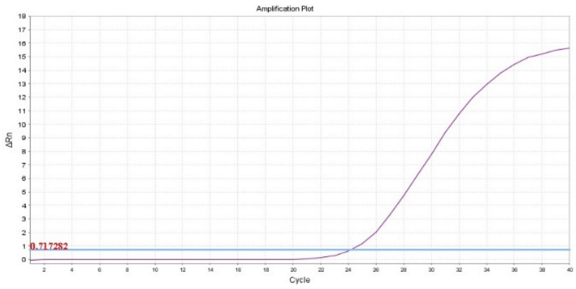

Control

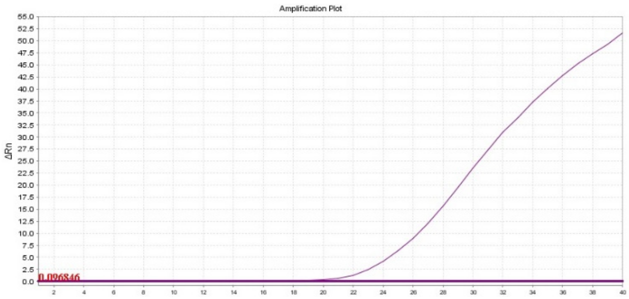

Standard

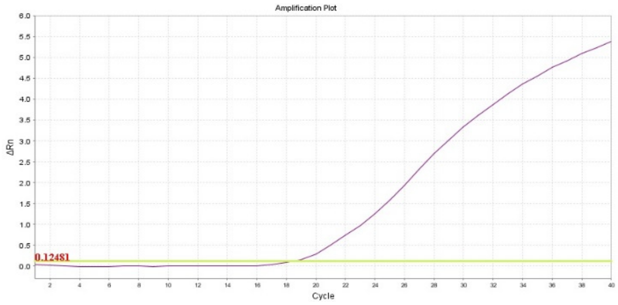

Hexane fraction

(e) qPCR curves obtained for p53 gene in treated MCF-7 cells

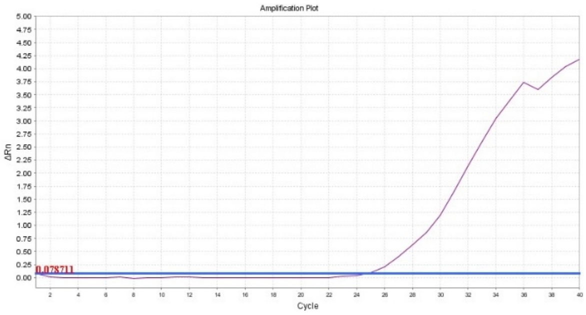

Control

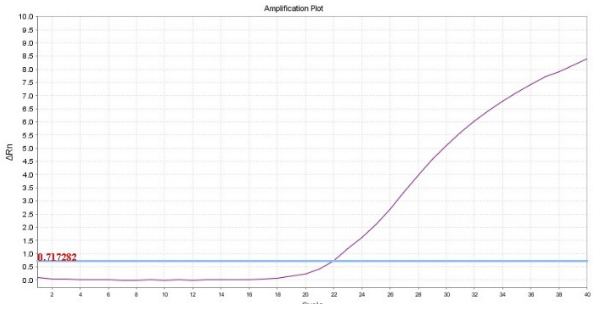

Standard

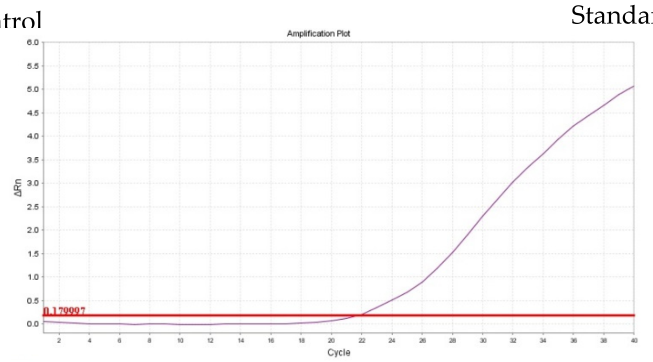

Hexane fraction

(f) qPCR curves obtained for p21 gene in treated MCF-7 cells

Figure S4. qPCR curves.
